# Supplementary material for: Identification and Evaluation of Olive Phenolics in the Context of Amine Oxidase Enzyme Inhibition and Depression: In Silico Modelling and In Vitro Validation
Source: Molecules. 2024 May 23;29(11):2446. doi: 10.3390/molecules29112446 (PMC11173677; doi:10.3390/molecules29112446)
Supplement: Supplementary file 1 [file molecules-29-02446-s001.zip › molecules-3018314-supplementary.pdf]

Identification and evaluation of olive phenolics in the context of amine oxidase enzyme inhibition and depression: *In silico* modelling and *in vitro* validation

Tom C. Karagiannis[1,2,3,4*], Katherine Ververis[2,3], Julia J. Liang[1,2,5], Eleni Pitsillou[2,5], Siyao Liu[6], Sarah M. Bresnehan[2], Vivian Xu[2], Stevano J. Wijoyo[2,7], Xiaofei Duan[8], Ken Ng[6], Andrew Hung[5], Erik Goebel[9], Assam El-Osta[1,7,10,11,12,13]

[1] Epigenetics in Human Health and Disease Program, Baker Heart and Diabetes Institute, 75 Commercial Road, Prahran, VIC 3004, Australia

[2] Epigenomic Medicine Laboratory at prospED Polytechnic, Carlton, VIC 3053, Australia

[3] Department of Clinical Pathology, The University of Melbourne, Parkville, VIC 3010, Australia

[4] Department of Microbiology and Immunology, The University of Melbourne, Parkville, VIC 3010, Australia

[5] School of Science, STEM College, RMIT University, VIC 3001, Australia

[6] School of Agriculture, Food and Ecosystem Sciences, Faculty of Science, The University of Melbourne, Parkville, VIC 3010, Australia

[7] Department of Diabetes, Central Clinical School, Monash University, Melbourne, VIC 3004, Australia

[8] Melbourne TrACEES Platform and School of Chemistry, Faculty of Science, The University of Melbourne, Parkville 3010, Australia

[9] Occhem Labs, LLC, 3510 Hopkins Place North, Oakdale, MN 55128, USA

[10] Department of Medicine and Therapeutics, The Chinese University of Hong Kong, Sha Tin, Hong Kong SAR

[11] Hong Kong Institute of Diabetes and Obesity, Prince of Wales Hospital, The Chinese University of Hong Kong, 3/F Lui Che Woo Clinical Sciences Building, 30-32 Ngan Shing Street, Sha Tin, Hong Kong SAR

[12] Li Ka Shing Institute of Health Sciences, The Chinese University of Hong Kong, Sha Tin, Hong Kong SAR

[13] Biomedical Laboratory Science, Department of Technology, Faculty of Health, University College Copenhagen, Copenhagen, Denmark

Short (running title): Oleohydroxypyretol and amine oxidase inhibition

Keywords: *Olea europaea*, olive phenolics, hydroxytyrosol, oleocanthal, oleohydroxypyretol, lysine-specific demethylase 1, monoamine oxidase

* Author for Correspondence:
Dr Tom Karagiannis
Epigenomic in Human Health and Disease Program
Baker Heart and Diabetes Institute
75 Commercial Road, Prahran, VIC 3004, Australia
Email: karat@unimelb.edu.au Phone: +613 8532 1290 Fax: +613 8532 1100

Table of Contents

Supplementary Methods.. 3

Table S1. Interactions between LSD1 and the histone H3 peptide in the substrate-binding cavity.. 12

Table S2. Predicted ligand-binding sites from the PrankWeb analysis are shown for LSD1 and MAO subtypes.. 13

Table S3. Growth factors and small molecules used for differentiation of hPSCs.......... 14

Table S4. Media components used for hESC-derived neurons cell line...................... 15

Table S5. Primary and secondary antibodies used in immunoblotting........................ 16

Table S6. List of primary and secondary antibodies used in Western blotting............... 17

Figure S1. Assessment of cell viability using the CellTiter-Blue® Assay kit............... 18

Figure S2. Viability of BJ cells.. 19

Figure S3. Potent inhibition of LSD1 by phenolic compounds.................................. 20

Figure S4. Phenolic compounds reduce LSD1 activity in BJ fibroblasts stimulated with DEX..21

Figure S5. Stimulation of MAO enzyme activity by HC within BJ cells......................22

Figure S6. Developmental pathway of neurons derived from hESCs..........................23

Figure S7. NMR spectra for the synthesised sample of OLP.................................. 24

Figure S8. LC-MS analysis for the synthesised sample of OLP.............................. 25

Figure S9. Upregulation of MAO protein expression by HC and DEX within BJ cells.... 26

Figure S10. Potent inhibition of MAO enzyme expression by OLP and OLC within BJ cells..27

Supplementary Methods

Synthesis of oleohydroxypyretol (OLP)

To validate our *in silico* studies, OLP was synthesised according to previously described protocols [38].

2-Trifluoroacetyloxysuccinic anhydride: Briefly, (R)-malic acid was placed into excess trifluoroacetic anhydride for 2 h. The mixture was concentrated under reduced pressure.

Methyl malic acid: The residue, 2-trifluoroacetylocysuccinic anhydride, (60.0 g, 283 mmol) was placed into methanol (200 mL) and stirred overnight. The mixture was then concentrated under reduced pressure and recrystallised from dichloromethane (DCM) (500 mL) to yield pure product (27.0 g, 64% yield).

tert-Butyldimethylsilyl (TBS)-protected methyl malic acid: Methyl malic acid (1.19 g, 8.04 mmol) was mixed with 50 mL of DCM and imidazole (1.64 g, 24.09 mmol). This was followed by the addition of tert-butyldimethylsilyl trifluoromethanesulfonate (TBS-OTf) dropwise (4.06 mL, 17.69 mmol) at 0° C and the mixture was allowed to come to room temperature overnight. Methanol was added (10 mL), the mixture was stirred for 10 minutes, and the mixture was concentrated under reduced pressure. Methanol (100 mL) was added, followed by potassium carbonate (1.3 g, 9.41 mmol), and the mixture was stirred for 2 h. The mixture was concentrated under reduced pressure and was taken up in ethyl acetate (100 mL), washed with 1M citric acid (50 mL), water (50 mL) and brine (50 mL). The organic layer was dried with Na_2SO_4 and concentrated under reduced pressure. The crude mixture was purified by silica gel column chromatography (2%-5% methanol in DCM) to give the product as a colourless solid after concentration (1.04 g, 63% yield).

Di-TBS hydroxytyrosol: Tri-TBS-3,4-dihydroxyphenylacetic acid (2.0 g, 3.9 mmol) was placed in tetrahydrofuran (THF) (100 mL). Lithium aluminum hydride (592 mg, 15.6 mmol) was added slowly at 0° C. The reaction was stirred for 1 h then diluted with 200 mL of diethyl ether. Water (1 mL) was added dropwise followed by NaOH (1 mL, 15 %) and water (3 mL). The mixture was warmed to room temperature and stirred for 15 minutes. $MgSO_4$ was added and stirred for 15 minutes, then filtered and concentrated under reduced pressure. The crude material was purified by silica gel chromatography (20 – 30 % EtOAc/Hexanes) to give Di-TBS hydroxytyrosol (1.19 g, 80 % yield).

Tri-TBS final product: TBS-methyl malic acid (1.00g, 3.86 mmol) was added to 100 mL DCM followed by (N,N′-dicyclohexylcarbodiimide) DCC (1.20 g, 5.70 mmol), Di-TBS hydroxytyrosol (1.48 g, 3.86 mmol), 4-dimethylaminopyridine (DMAP) (140 mg, 1.14 mmol) and triethylamine (536 uL, 3.86 mmol). The mixture was stirred overnight, then washed with 50 mL water, 50 mL brine, dried with Na_2SO_4 and concentrated under reduced pressure. The crude product was purified with silica gel column chromatography (conditions) to give product (875 mg, 36% yield).

OLP: Tetrabutyl ammonium fluoride (24 mL, 1 M in THF, 24 mmol) was added dropwise to the product (4.30 g, 6.86 mmol) in 70 mL THF. The mixture was stirred for 15 minutes after which thin layer chromatography (TLC) indicated the reaction was complete. The mixture was poured into 250 mL water and extracted with ethyl acetate (EtOAc) (200 x 3). Organic layer was washed with brine and dried with Na_2SO_4 and was concentrated under reduced pressure to yield 3.42 g crude. Column chromatography 50% EtOAc/Hexanes; 100 % EtOAc yielded 1.725 g (89% yield) of OLP after evaporation.

NMR and LC-MS analysis of OLP

NMR was performed to characterise the structure of OLP using ^{1}H, ^{13}C, and ^{13}C DEPT-135 (Bruker 400MHz Avance equipped with iProbe). OLP was dissolved in DMSO with final concentration of 0.5 mg/mL and 5 µL was injected into a HPLC-DAD-ESI-MS system (Agilent 1260 Infinity II LC MSD, Australia). Separation was carried out with Phenomenex Gemini C18 reverse phase column (California, United States) with mobile phase A (0.1% v/v formic acid in water) and B (0.1%v/v formic acid in acetonitrile), and gradient set up as follows: 5% B (0 – 2min), 30% B (2 – 10 min), 35% B (10 – 15 min), 40% B (15 – 20 min), 45% B (20 – 25 min), 50% B (25 – 30 min), 60% B (30 – 35 min), 100% B (35 – 45 min), at a flow rate of 0.8 mL/min. The acquisition wavelengths of the DAD were set at 280 nm. ESI chamber was set up with gas temperature of 325 °C, drying gas flow of 9.0 L/min, and capillary voltage of 3500V to produce [M-H]- adducts. Mass spectrometer detector (MS) was set at mass range of 100.00 – 1000.00 with negative mode scanning. OLP was identified as the major peak in DAD signal and meanwhile the major peak containing OLP product ion in total ion chromatogram (TIC) with same retention time.

BJ cells: MAO expression

Protein extraction

Growth medium was removed and a vortex (Scientific Industries SI-0236, NY USA) was used to disrupt the cell pellet. Cell solution was suspended in 1.5 mL of ice-cold D-PBS and aliquoted into a 1.5 mL eppendorf tube. The tubes were centrifuged at 4°C on 16000g for 10 minutes. The supernatant was then collected and transferred into fresh tubes. M-PER (Thermo Fisher Scientific, Thermo Scientific 78501, MA USA) (at a ratio of 60 μL to every 1 x 10^6 cells) was added along with 2.4 μL of protease inhibitor (Thermo Fisher Scientific, Thermo Scientific A32963, MA USA).

Bradford assay

Protein samples were thawed on ice and a protein concentration plate was prepared to a 1:5 dilution and 1:10 dilution (4μL of sample to 16μL of nuclease-free H_2O and 5μL of 1:5 dilution sample with 5μL of nuclease free H_2O) (Thermo Fisher Scientific, Invitrogen AM9932, MA USA). 10μL of each dilution preparation was pipetted into wells of a 96-well assay microplate (Sigma-Aldrich, Corning CLS3997, MO USA). At a range from 0-2000μg/mL, 10μL of pre-prepared BSA Albumin Standard dilutions (Thermo Fisher Scientific, Thermo Scientific 23209, MA USA) were transferred into assigned wells. Coomassie Blue Bradford Reagent (250μL) (Sigma-Aldrich B6916, MO USA) was then pipetted into all wells. The microplate was covered in foil and left to incubate at room temperature for 10 minutes with gentle shaking on an orbital mixer (Ratek EOM5, Melbourne, Australia). Using a CLARIOstar Microplate Reader (BMG Labtech, Ortenberg, Germany), the absorbance of the microplate was measured at 595nm.

Gel electrophoresis

Combs were removed from pre-cast 12-well Bolt 4-12% Bis-Tris Plus gels (Thermo Fisher Scientific, Invitrogen NW04120BOX, MA USA). Gels in each chamber were put into place via a clamp and 1X NuPAGE MOPS SDS running buffer (0.1% SDS (w/v), 50mM MOPs, 50mMTris base, 1mM EDTA) was dispensed two thirds full into the XCell SureLock Mini-Cell Electrophoresis System (Thermo Fisher Scientific, Invitrogen EI0001, MA USA). Prior to loading, protein samples were heated to 70°C for 10 minutes on a Block Digital Dry Block Heater (Ratek DBH10DP) and wells were washed with running buffer using a 21G syringe (Terumo Corporation, Laguna, Philippines). 6μL of protein standard (SeeBlue Plus2) (Thermo Fisher Scientific, Invitrogen LC5925, MA USA) was loaded into a well preceding protein sample. Gels ran at 200V for approximately 30 minutes. Protein was loaded within a concentration range of 10- 50μg/well along with 10μL of TruPAGE™ LDS Sample Buffer

(Sigma-Aldrich PCG3009).

Membrane transfer

Gels were transferred onto a Transblot Turbo Mini Size LF PVDF Membrane (Bio-Rad Laboratories Inc 10026934, CA USA) by pre-soaking labelled membranes in 100% methanol (Sigma-Aldrich 322415). Membranes, along with Trans- Blot Turbo Mini-size Transfer Stacks **(**Bio-Rad Laboratories Inc 10026930, CA USA) were soaked in NuPAGE transfer buffer (pH 7.2, 25mM Bicine, 25mM Bis-tris, 0.05mM Chlorobutanol, 1.0mM EDTA). Assembled within a Trans-Blot Turbo Transfer System (Bio-Rad Laboratories Inc 1704150, CA USA), the gel was layered into a cassette with blotting membrane sandwiched between wet transfer stacks. Using a blot roller (Bio-Rad Laboratories Inc 1651279, CA USA), air bubbles were removed. The transfer system was set to 1.3A, 25V and let to run for 10 minutes. Following transfer, the membrane was air dried and the remaining stack was discarded.

Immunoblotting and Odyssey imaging

In preparation for imaging, dried membranes were washed in 100% methanol followed by Milli-Q H_2O. Membranes were then incubated in 5mL of REVERT Total Protein Stain (LI-COR 926-11011, NE USA) on an orbital mixer for 5 minutes and then with REVERT wash solution (30% (v/v) methanol (Sigma-Aldrich 322415-1L) and 6.7% (v/v) glacial acetic acid (Sigma-Aldrich 45754) for 1 minute. Prior to imaging, membranes were rinsed in Milli-Q H_2O. Using the Odyssey Clx Imaging System (LI-COR, NE USA), membranes were imaged immediately at 700nm. The REVERT stain was then stripped by incubating membranes with REVERT reversal solution (30% methanol (v/v) and 0.1% (w/v) NaOH)) for 5 minutes.

Membranes were washed in Milli-Q H_2O and blocked for one hour with gentle shaking on an orbital mixer at room temperature using Odyssey Blocking Buffer (LI-COR 927-50000, NE USA). Primary antibodies were diluted into a solution using 1:500-1:2000 (v/v) dilution ratios (primary antibody to Odyssey Blocking Buffer). Following a rinse with PBST (1XPBS, 0.05% Tween 20 (Sigma-Aldrich P1379, v/v) membranes were incubated with primary antibodies for 24 hours at 4°C on an orbital mixer. After 24 hours, the antibody solution was removed, and membranes were washed in PBST. An appropriate IRDye secondary antibody was prepared in Odyssey Blocking Buffer where membranes were immersed for 1 hour at room temperature. Before imaging, membranes were rinsed in both PBST and 1X PBS. Wavelength channels were set corresponding to the appropriate IRDye secondary antibody used. Protein quantification and expression analysis was performed using Image Studio Lite

Version 5 (LI-COR, NE USA).

BJ cells: LSD1 activity and H3K4 methylation status

Mammalian protein extraction

Growth medium was carefully decanted from T-75 culture flasks and briefly washed with PBS (1X) (137mM NaCl, 2.7mM KCl, 10mM Na2HPO4, 1.8mM KH2PO4). 8mL of fresh PBS was then added to the flask and cells were detached via gentle mechanical disruption using a cell scraper. Cell solution was then transferred into a 15mL and centrifuged at 335g for 5 minutes. After removing all PBS (1X) the cell pellet was broken via tapping, then resuspended in 1mL of ice cold PBS (1X) and transferred to a 1.5mL microcentrifuge tube. Cells were then centrifuged at 95g for 10 minutes at 4ºC. Supernatant was then removed via careful aspiration and the required amount of MPER (ThermoFisher Scientific 78501, MA USA) + Pi (Roche 11836153001, Mannheim Germany) was added to the cell pellet. For every 1 x 106 cells, 60µL of MPER (+2.4µL Pi) was added.

Cells were then lysed via a 15 minute agitation at 4ºC, and centrifuged for a final time at 14,000g for 15 minutes at 4ºC to separate cellular debris from the soluble protein fraction. Supernatant containing the protein soluble fraction was then transferred to a fresh 0.5mL microcentrifuge tube and stored at -80ºC. Remainder cell debris pellet was discarded.

Nuclear/cytosol protein fractionation

A nuclear/cytosol fractionation kit (BioVision K266-25, CA USA) was used to obtain separation of nuclear extract from cytoplasmic fraction of BJ cells. Cells were detached from flask surface via gentle mechanical disruption using a cell scraper. Cell solution was then transferred into a 15mL tube and centrifuged at 335g for 5 minutes. Growth medium was then removed via decanting and aspiration, with the remaining cell pellet resuspended in 1mL ice cold PBS (1X). Cell solution was then transferred into a 1.5mL microcentrifuge tube and spun at 600g for 5 minutes at 4°C. For every 2 x 10^6 cells, 0.2mL of Cytosol Extraction Buffer-A (CEB-A) mix, pre-prepared with 0.2µL 1M Dithiothreitol (DTT) and 0.4µL Protease Inhibitor Cocktail (PIC), was added. Cells were vortexed vigorously for 15 minutes to fully resuspend the cell pellet, followed by a 10 minute incubation on ice. 11µL of pre-chilled Cytosol Extraction Buffer B (CEB-B) was then added, after which each tube was vortexed at the highest setting for two separate 5 second periods. This was immediately followed by a 5 minute 16,000g centrifugation at 4°C. The resulting supernatant containing the cytoplasmic extract fraction was transferred to clean 0.5mL tubes and stored at -80°C.

100μL of Nuclear Extraction Buffer containing 0.2μL 1M DTT and 0.4μL PIC was then added to the remaining nuclei-containing pellets. Full resuspension was achieved through multiple rounds of vigorous vortexing and ice incubations (to maintain protein integrity). Once the entire pellet was visibly resuspended in NEB, tubes were centrifuged at 16,000g for 10 minutes. The resulting supernatant containing the nuclear extract fraction was transferred to clean 0.5mL tubes and stored at -80°C.

Acid extraction of histone proteins

Cells were washed in 10mL of pre-chilled PBS (1X) and detached from flask surface via mechanical disruption using a cell scraper. Cell solution was then transferred into a 15mL tube and centrifuged at 150g for 10 minutes. Supernatant was then discarded and the remaining cell pellet resuspended in 0.5mL PBS (1X) and transferred into a 1.5mL microcentrifuge tube. Cells were then centrifuged at 9400g for 20 seconds, after which the supernatant was removed and discarded. The remaining pellet was then briefly resuspended in 600μL of acid extraction lysis buffer (10mM KCl, 10mM HEPES, 1.5nM $MgCl_2$) containing cOmplete™ Mini Protease Inhibitor Cocktail (Sigma-Aldrich, Roche 11836153001, MO USA) and 0.3μL 1M DTT. This will cause cells to pre-swell, resulting in a reduced amount of PBS (1X) remaining in the microcentrifuge tube that may deter maximal cell lysis capacity. Cells were then centrifuged at 9400g for 20 seconds with the resultant supernatant aspirated and discarded.

Cells were then lysed by adding 250μL of acid extraction lysis buffer containing cOmplete™ Mini Protease Inhibitor Cocktail, followed by vigorous vortexing to achieve complete resuspension. 16.25μL of Sulphuric acid (Sigma-Aldrich 339741, MO USA) was then added to the sample and vigorously vortexed to evenly disperse the acid. Sample containing tubes were then left to incubate on ice for 1 hour with intermittent vortexing every 15 minutes. Remaining cellular debris was removed through centrifugation at 15,870g at 4°C for 10 minutes. The supernatant fraction containing acid soluble proteins were transferred into a clean 1.5mL tube, in which acid soluble proteins were precipitated with 9 volumes of acetone (Sigma-Aldrich 650501, MO USA) at -20°C for 1 hour. To obtain resultant precipitate in pellet form, samples were centrifuged at 15,870g for 10 minutes at 4°C and supernatant was discarded thereafter. Protein pellets were then washed in 70% ethanol, air-dried and dissolved in 60μL of Milli-Q H_2O (Merck Millipore, MA USA). Maximal dissolution was achieved through intermittent pipetting over a 1-2 hour on-ice incubation period. The resultant solution

containing desired acid soluble histone proteins were stored at -80°C.

Histone demethylase LSD1 activity assay

4μL of nuclear extracts containing 5μg protein was added to designated strip wells with stably captured LSD1 substrate (H3K4me2), followed by an additional 26μL of H3K4me2 (HG3) substrate diluted in assay buffer (HG2). In no enzyme control wells, the 4μL of nuclear extracts was replaced with 4μL of assay buffer. Blank wells contained 30μL of assay buffer. A standard curve was generated by adding 1μL of standard (HG4) at incrementally increasing concentrations (0.1-10ng/μL) to 29μL of assay buffer in designated standard wells. After all wells were loaded appropriately, the assay plate was incubated away from light at room temperature for 60 minutes. Following incubation, contents were aspirated and wells were washed three times with 150μL of wash buffer (HG1). 50μL of capture antibody (HG5) diluted at a 1:1000 ratio was then added to all wells before incubating at room temperature for 60 minutes on a rotating platform. Well contents were then aspirated and washed four times with 150μL of wash buffer. 50μL of detection antibody (HG6) diluted at a 1:1000 ration was then added to each well. Assay plate was then incubated for a final time at room temperature for 30 minutes. Well contents were aspirated again, followed by six washes with 150μL wash buffer. 50μL of fluoro-developer (HG7) was then added into each well and allowed to develop away from light for 1-5 minutes. Fluorescence was then read on a CLARIOstar Microplate Reader (BMG Labtech, Ortenberg Germany) at Ex/Em = 530/590nm.

Bradford Assay

The Bradford assay was performed as previously described. Histone protein samples obtained from BJ cells were prepared for total loading volume of 22μL at a protein concentration of 1μg per well. Cytosolic protein fractions obtained from BJ cells were prepared for a total loading volume of 40μL at a protein concentration of 10μg per well. All final volumes of prepared loading sample is inclusive of 5μL NuPAGE LDS Sample Buffer (4X) (Thermo Fisher Scientific, Invitrogen NP0007, MA USA) used for sample weight.

Gel electrophoresis

Pre-prepared samples were heated on a dry block heater at 95°C for 5 minutes to decrease viscosity. Gel combs were removed from 10 or 15 well Bolt 4-12% Bis-Tris Plus Gels (Thermo Fisher Scientific, Invitrogen NW04120BOX (10 well)/BG04125BOX (15 well), MA USA). All gels were run in an XCell SureLock Mini-Cell Electrophoresis System (Thermo Fisher

Scientific, Invitrogen EI0001, MA USA). Gels in each mini-chamber were clamped into place and 1x NuPAGE MOPS SDS running buffer (50nM MOPS, 50mM Tris base, 0.1% SDS, 1mM EDTA, pH 7.7) was poured into the central chamber to 2/3^{rds} maximal volume. Each well was washed twice with running buffer using a 22 gauge syringe (Terumo Corporation, Laguna Philippines) after which 6μL of SeeBlue Plus2 pre-stained protein standard (Thermo Fisher Scientific, Invitrogen LC5925, MA USA) was loaded into the first well followed by test samples at their appropriate loading volumes. Gels were run at 200V for an initial 10 minutes, then at 150V for approx. 45-90 minutes.

Membrane transfer

Gels were then transferred onto Odyssey Imaging System-compatible PVDF Immobilon-FL transfer membranes (Sigma-Aldrich, Millipore IPFL00010, MO USA) pre-soaked in 100% methanol (Sigma-Aldrich 322415, MO USA) for 1 minute. Constructed transfer chambers were then placed in a mini cell tank (Thermo Fisher Scientific, Invitrogen, MA USA) and filled with enough NuPAGE transfer buffer (25mM Bicine, 25mM Bis-tris, 1.0mM EDTA, 0.05mM Chlorobutanol, pH 7.2) to cover the blotting pads. Mini tanks were then left to transfer overnight at 30V.

Immunoblotting and Odyssey imaging

An initial total protein stain was carried out to ensure gel-to-membrane transfer was successful, as well as for use in expression analysis and normalisation. After removal from transfer chambers, membranes were air-dried, soaked briefly in 100% methanol and rinsed with Milli-Q H_2O. Membranes were then incubated on a rotating platform with 5mL of REVERT Total Protein Stain (LI-COR 926-11011, NE USA). Membranes were then washed in REVERT wash solution (6.7% glacial acetic acid, 30% (v/v) methanol) and Milli-Q H_2O, and immediately imaged at 700nm using the Odyssey CLx Imaging System. Stain was then removed with REVERT reversal solution (0.1% (w/v) NaOH, 30% (v/v) methanol) and rinsed with Milli-Q H_2O before proceeding to blocking and immunodetection.

Membranes were blocked in Odyssey Blocking Buffer (LI-COR 927-50000, NE USA) for 1 hour at room temperature on a rotating platform followed by a brief rinse in PBST. Primary antibodies were made up in Odyssey Blocking Buffer to their appropriate dilution ratios (as specified by manufacturers' protocol) ranging from 1:500-1:2000 (v/v). Membranes were then incubated in primary antibody overnight at 4°C. Following removal of primary antibody, membranes were washed three times in PBST before a 1 hour incubation with appropriate

IRDye secondary antibody secondary antibody at room temperature. Membranes were then washed again with PBST three times followed by a brief rinse in 1xPBS.

Membranes were imaged with the Odyssey CLx Imaging Workstation (LI-COR, NE USA) using appropriate channels (800nm) as indicated by IRDye secondary antibody specifications. Expression analysis and quantification was carried out using Image Studio Lite Version 5.2 (LI-COR, NE USA).

Table S1. Interactions between LSD1 and the histone H3 peptide in the substrate-binding cavity.

	Crystal structure		OLC bound to LSD1	
	Peptide	LSD1	Peptide	LSD1
**Hydrogen bonds**	ALA 1	ALA 539	ALA 1	ALA 539
	ARG 2	ASP 556	ARG 2	ASP 556
	ARG 2	ASP 553	ARG 2	ASP 553
	THR 3	ASP 555	THR 3	ASP 556
	GLN 5	ASN 535	THR 3	ASP 555
	ARG 8	ASP 375	GLN 5	ASN 535
	ARG 8	GLU 379	ARG 8	ASP 375
	ARG 8	ASP 375	THR 11	ASN 383
	ARG 8	CYS 360	GLY 12	ASN 383
	THR 11	ASN 383	LYS 14	GLU 559
	GLY 12	ASN 383	THR 6	HIS 564
	LYS 14	GLU 559		
	THR 6	HIS 564		
**Salt bridges**	ARG 2	ASP 556	ARG 2	ASP 556
	ARG 2	ASP 556	ARG 2	ASP 556
	ARG 2	ASP 553	ARG 2	ASP 553
	ARG 2	ASP 553	ARG 2	ASP 553
	ARG 8	GLU 379	ARG 8	ASP 375
	ARG 8	ASP 375	LYS 14	GLU 559
	ARG 8	ASP 375		
	ARG 8	GLU 379		
	ARG 8	ASP 375		
	ARG 8	ASP 375		
	LYS 14	GLU 559		

Table S2. Predicted ligand-binding sites from the PrankWeb analysis are shown for LSD1 and MAO subtypes.

Protein	Ligandability Score	Residues
LSD1	16.35	284, 285, 287, 288, 289, 307, 308, 309 ,310 ,315, 316, 317, 331, 588, 589, 590, 591, 624 ,625, 626, 629, 636, 637, 756, 761, 800, 801, 802, 809, 810, 811, 814
MAO-A	35.45	111, 180, 181, 19, 20, 207, 208, 209, 210, 214, 215, 22, 23, 24, 243, 244, 272, 273, 274, 277, 280, 303, 305, 323, 325, 335, 337, 350, 352, 401, 402, 403, 407, 42, 43, 434, 435, 436, 44, 443, 444, 445, 448, 49, 50, 51, 52, 66, 67, 68, 69, 93, 97
MAO-B	Chain A: 52.22	10, 102, 104, 11, 119, 13, 14, 164, 167, 168, 171, 172, 198, 199, 201, 206, 234, 235, 263, 264, 265, 268, 271, 272, 294, 296, 316, 326, 34, 343, 35, 388, 393, 397, 398, 40, 41, 42, 425, 426, 43, 434, 435, 436, 439, 57, 58, 59, 60, 84, 88
	Chain B: 57.05	10, 102, 104, 11, 119, 12, 13, 14, 15, 164, 167, 168, 171, 172, 188, 198, 199, 201, 206, 235, 263, 264, 265, 268, 271, 272 ,296, 316 ,326, 328, 33, 34, 343, 35, 36, 388, 393, 394, 397, 398, 40, 41, 42, 426, 427, 43, 434, 435, 436, 439, 57, 58, 59, 60, 84, 88

Table S3. Growth factors and small molecules used for differentiation of hPSCs.

Type	Name	Concentration	Supplier	Serial/Catalogue Number
Small molecules	SB431542 (SB)	10 mM	Miltenyi Biotec	SB431542
	LDN 193189	100 nM	KareBay Biochem	KI1121
Growth factors	Epidermal Growth Factor (EGF)	20 ng/mL	Thermo Fisher Scientific, Gibco	AF-100-15-100UG
	Basic-Fibroblast Growth Factor (bFGF)	20 ng/mL	Thermo Fisher Scientific, Gibco	100-18B-100UG

Table S4. Media components used for hESC-derived neurons cell line.

Media	Component	Concentration of stock	Volume in mL/500 mL	Supplier	Catalogue Number
**NBM**	N2	1	5	Thermo Fisher Scientific, Gibco	17502048
	Neurobasal Medium	-	470	Thermo Fisher Scientific, Gibco	21103049
	B27 minus vitamin A	1	10	Thermo Fisher Scientific, Gibco	12587010
	Insulin-Transferrin-Selenium-Sodium Pyruvate	1	5	Thermo Fisher Scientific, Gibco	51300044
	GlutaMAX	2 mM	5	Thermo Fisher Scientific, Gibco	35050061
	Penicillin-Streptomycin	1	5	Thermo Fisher Scientific, Gibco	15140122

Table S5. Primary and secondary antibodies used in immunoblotting.

	Antibody	Species	Dilution Factor	Supplier	Serial/Catalogue Number
**Primary**	Monoamine oxidase A	Rabbit	1:5000	Abcam	ab126751
	Monoamine oxidase B	Rabbit	1:5000	Abcam	ab175136
	GAPDH	Mouse	1:2000	Abcam	ab9484
**Secondary**	IRDye anti-mouse	Donkey	1:20000	LI-COR	926-68072
	IRDye anti-rabbit	Goat	1:20000	LI-COR	925-32211

Table S6. List of primary and secondary antibodies used in Western blotting.

Antibody	Species	Manufacturer	Catalogue No.	Dilution Factor
H3K4me1	Rabbit	Abcam	ab176877	1:5000
IRDye Donkey anti-Rabbit (Secondary)	Donkey	LI-COR	926-32213	1:2000

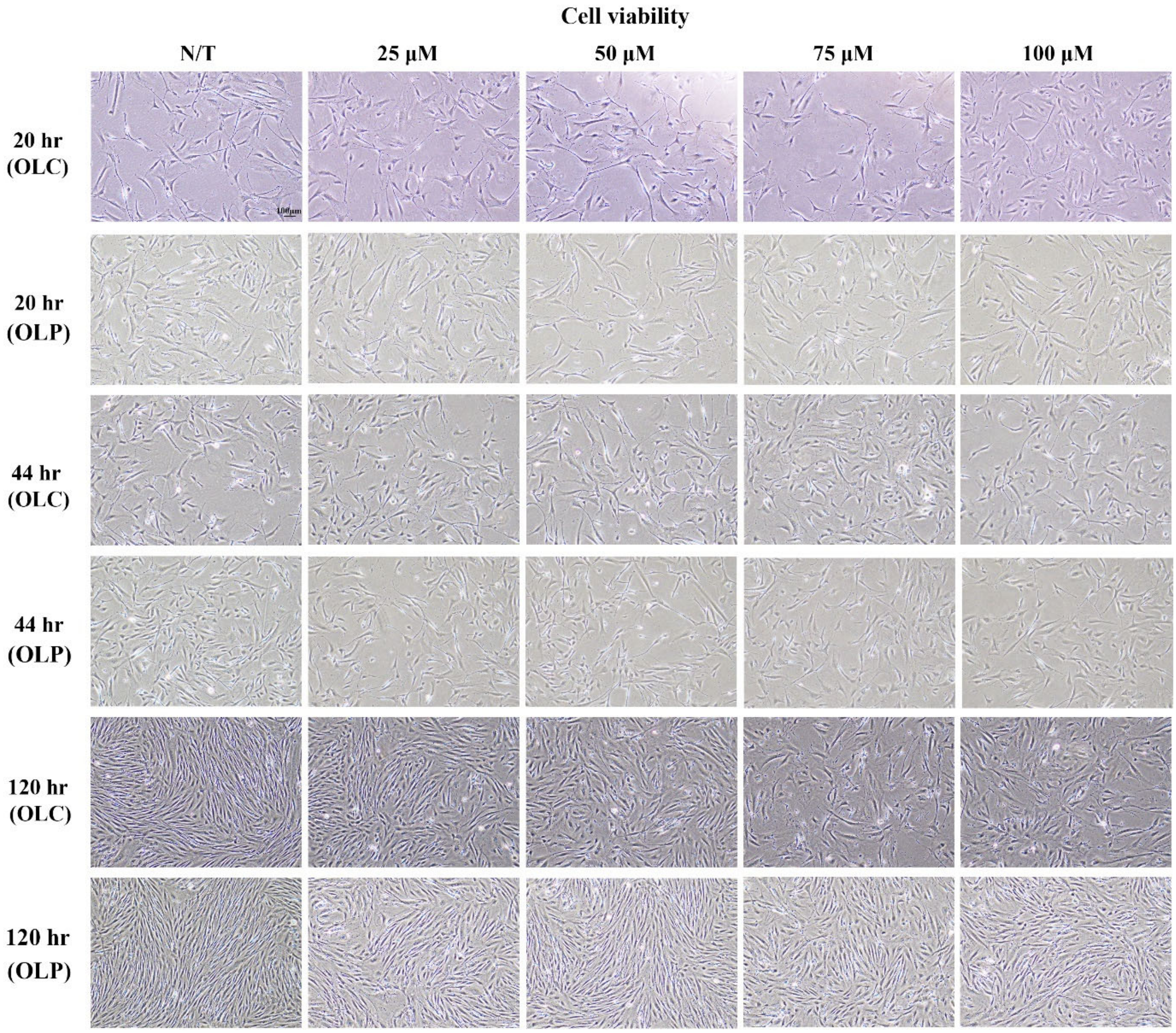

Figure S1. Assessment of cell viability using the CellTiter-Blue® Assay kit. BJ cells were either untreated (N/T) or treated with increasing concentrations (25, 50, 75, and 100 µM) of OLC and OLP. BJ cells were imaged at 4 x objective magnification at 20, 44, and 120 h using a Nikon Eclipse Ts2 light microscope.

A

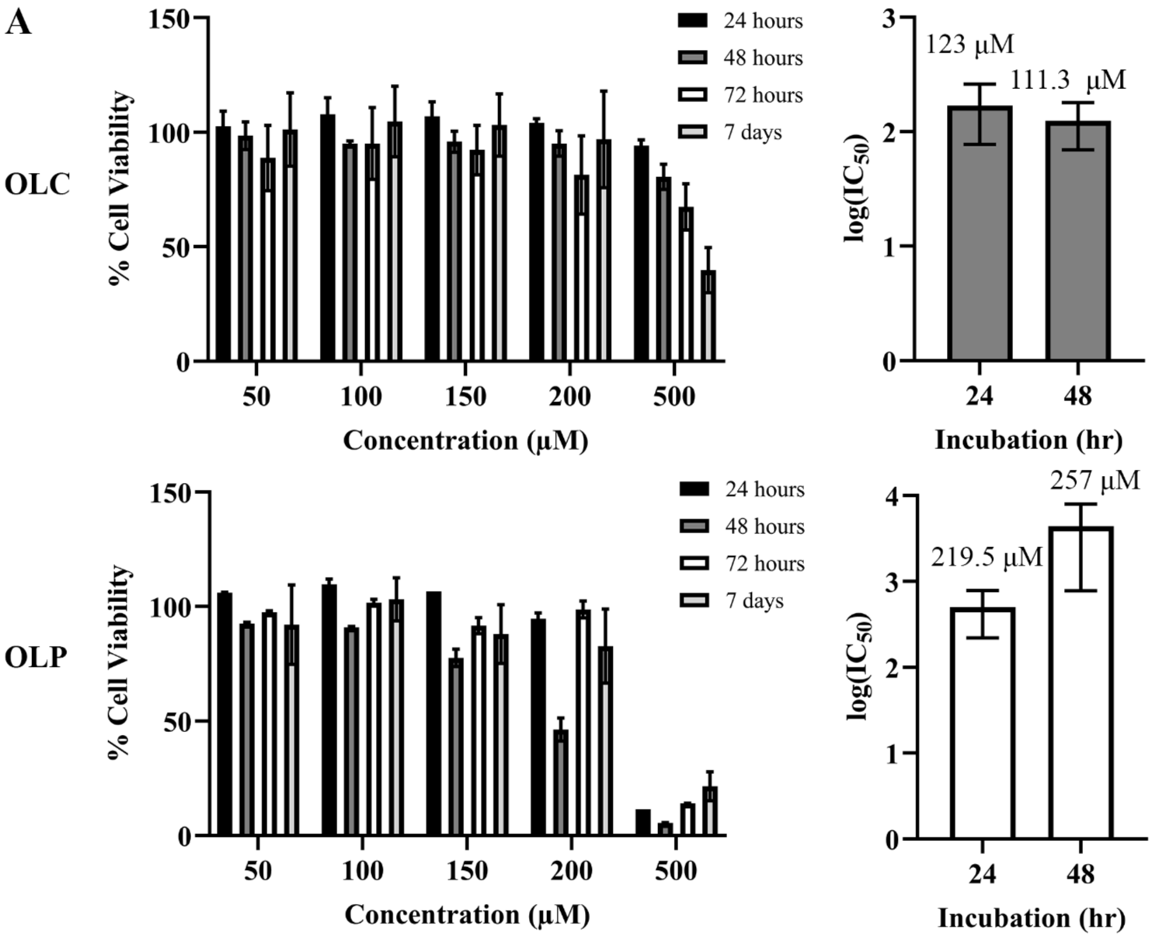

Figure S2. Viability of BJ cells. (A) The relative cell viability (%) of BJ cells treated with 0-500 µM OLC and OLP was measured at 24 h, 48 h, 72 h, and 7 days. The results are shown for 50, 100, 150, 200, and 500 µM. Error bars represent the % SEM from duplicate assays. The IC_{50} values were calculated at 24 h and 48 h. Data presented denotes the mean ± SEM from duplicate assays (representative results from n = 3 independent experiments).

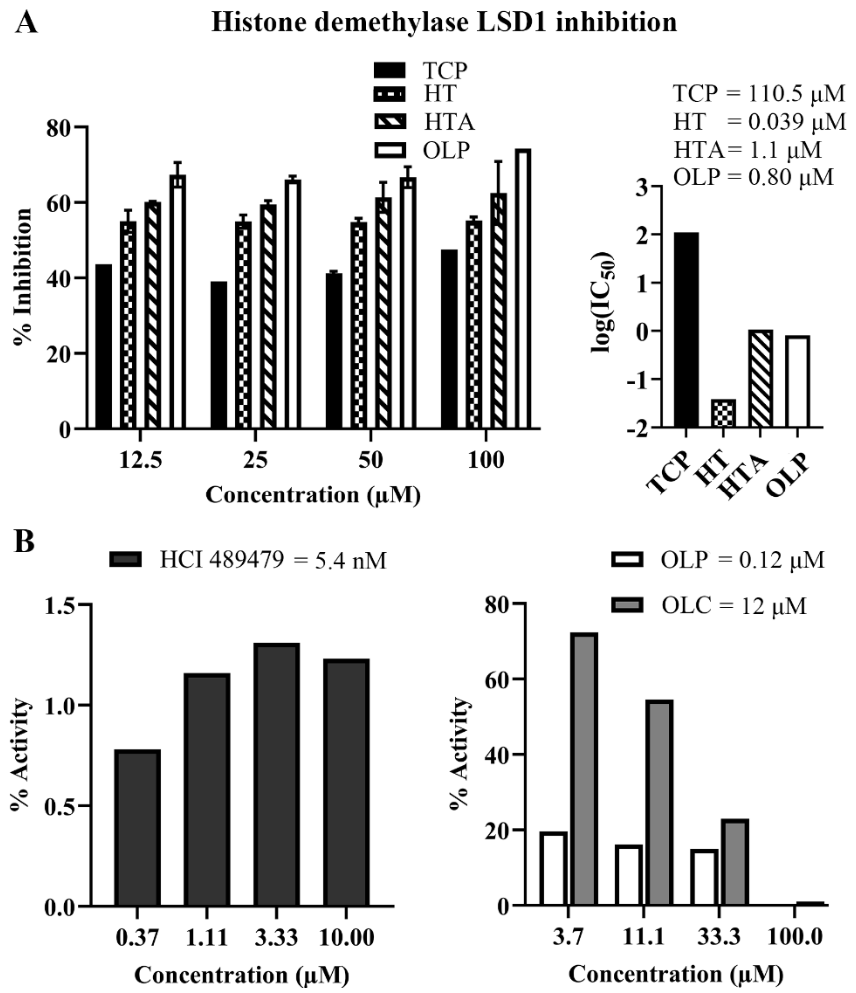

Figure S3. Potent inhibition of LSD1 by phenolic compounds. (A) Inhibition (%) of LSD1 by the control compound TCP and the phenolic compounds HT, HTA, and OLP at concentrations of 12.5, 25, 50, and 100 μM. Data presented denotes the mean ± SEM from duplicate (TCP, HTA, OLP) and triplicate (HT) assays (representative results from n = 3 independent experiments). A nonlinear regression analysis was performed to determine the IC_{50} values: TCP = 110.5 μM, HT = 0.039 μM, HTA = 1.1 μM, and OLP = 0.80 μM. (B) LSD1 was pre-incubated with the control compound HCl 489479, OLP, and OLC prior to the addition of the peptide substrate. The demethylase activity (%) of LSD1 was measured for the control compound HCl 489479 and the phenolic compounds (OLP and OLC) at concentrations ranging from 0-10 μM and 0-100 μM, respectively. The IC_{50} values were determined to be 5.4 nM, 12 μM, and 0.12 μM for HCl 489479, OLC, and OLP, respectively (as performed by Reaction Biology Corporation, fluorescence coupling enzyme assay).

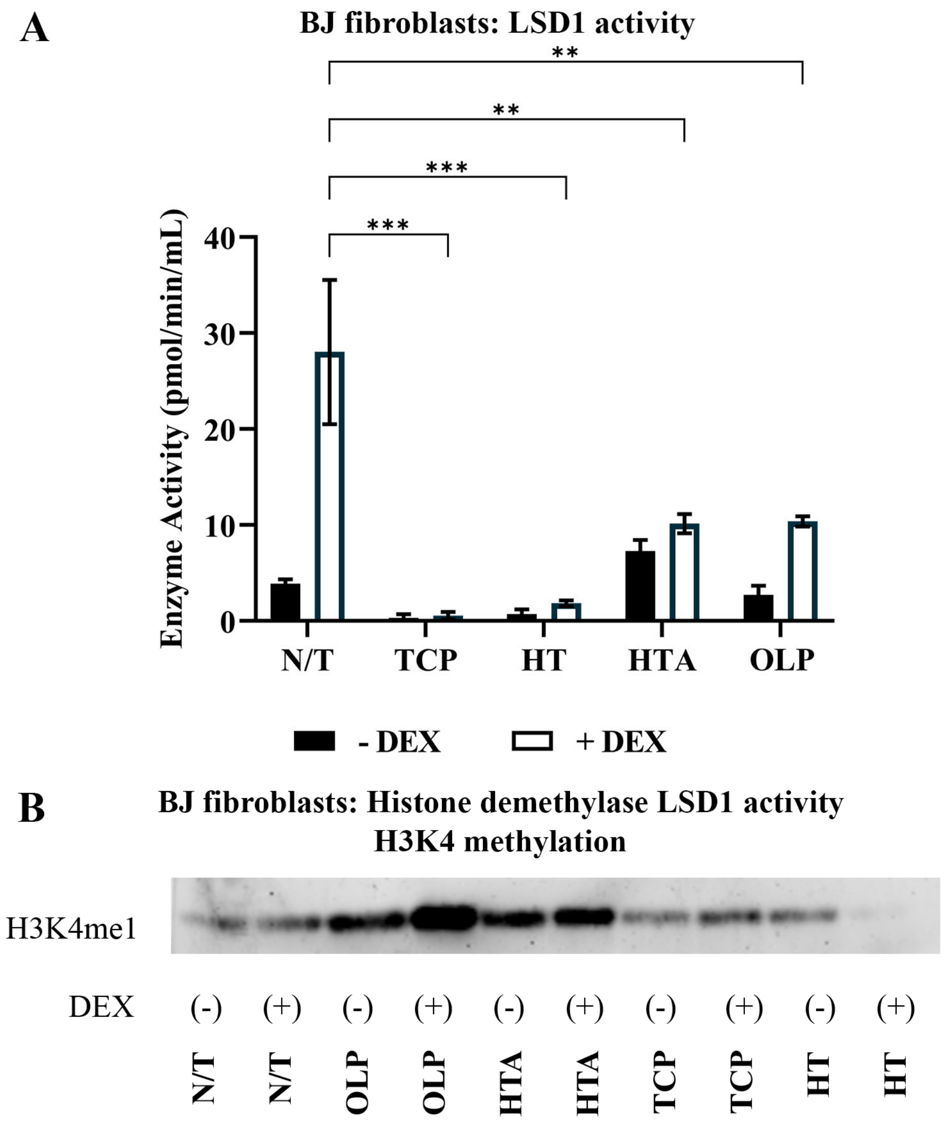

Figure S4. Phenolic compounds reduce LSD1 activity in BJ fibroblasts stimulated with DEX. (A) BJ cell cultures were stimulated with DEX (50 μM) or incubated with normal growth medium for 72 h prior to treatment with the established inhibitor TCP (5 μM) or the phenolic compounds OLP (50 μM), HT (50 μM), and HTA (50 μM) for 48 h. (+ and – is indicative of DEX treatment only; type of test inhibitor treatment is stated in the axis). Nuclear proteins extracted from treated BJ cultures were directly assayed. (B) Histone proteins extracted from treated cultures were analysed through Western blotting, where methylation status was determined via immunodetection via mono-methylated lysines on histone H3.

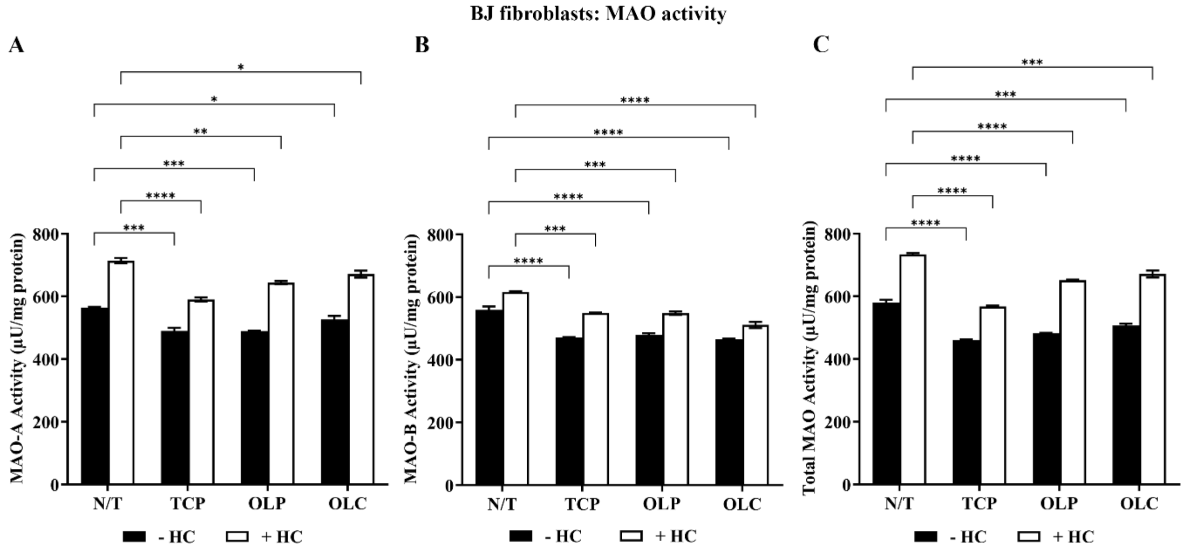

Figure S5. Stimulation of MAO enzyme activity by HC within BJ cells. (A-C) BJ cells were incubated with normal growth medium (-HC) or 10 µM HC (+HC) for 5 days and treated with 50 µM OLP, 50 µM OLC, or 5µM TCP for 24 h . The results depict the activity of (A) MAO-A, (B) MAO-B, and the (C) total MAO activity. Data obtained is represented as the mean ± SEM from duplicate assays. *$p \leq 0.05$, **$p \leq 0.01$, *** $p \leq 0.001$, **** $p \leq 0.0001$ quantified using a 2-way ANOVA with Tukey's post-hoc multiple comparisons test.

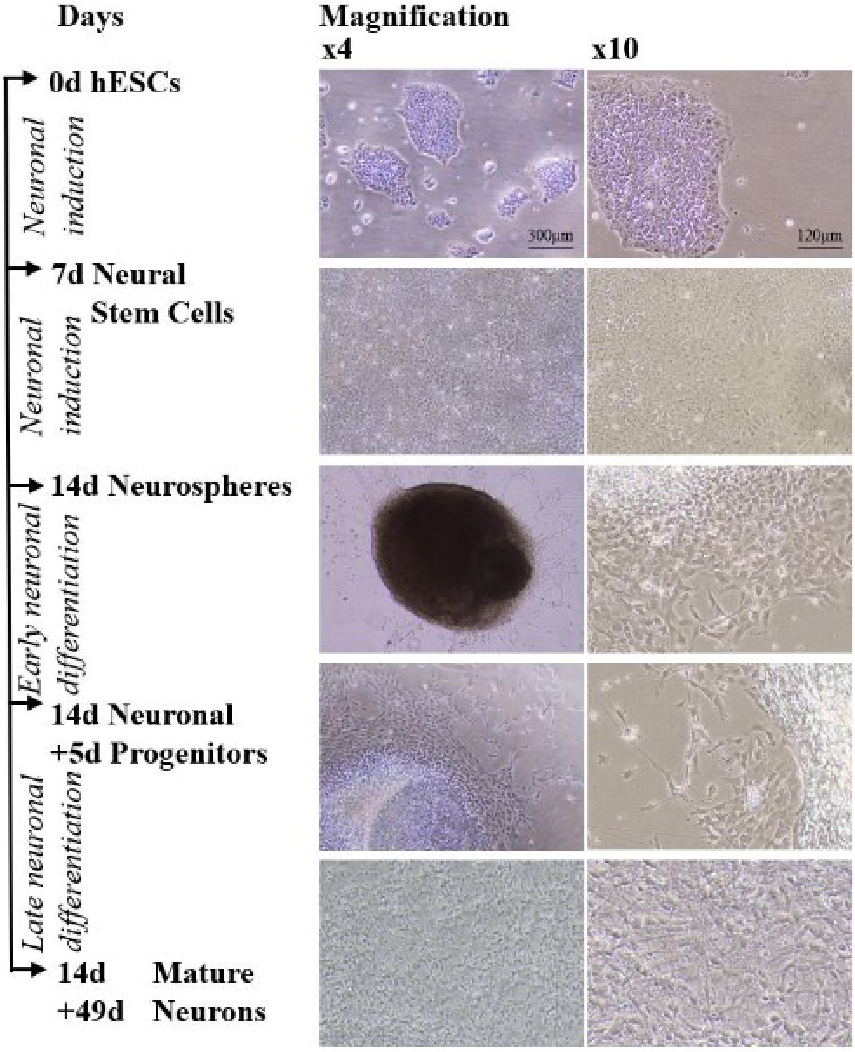

Figure S6. Developmental pathway of neurons derived from hESCs. Cells were incubated with EGF and bFGF to induce neuron-like characteristics. Following neurosphere formation, cells appeared as progenitors (+19d) where half the total volume of NBM media from wells was replaced every 3 days to promote late neuronal differentiation and transformation into mature neurons.

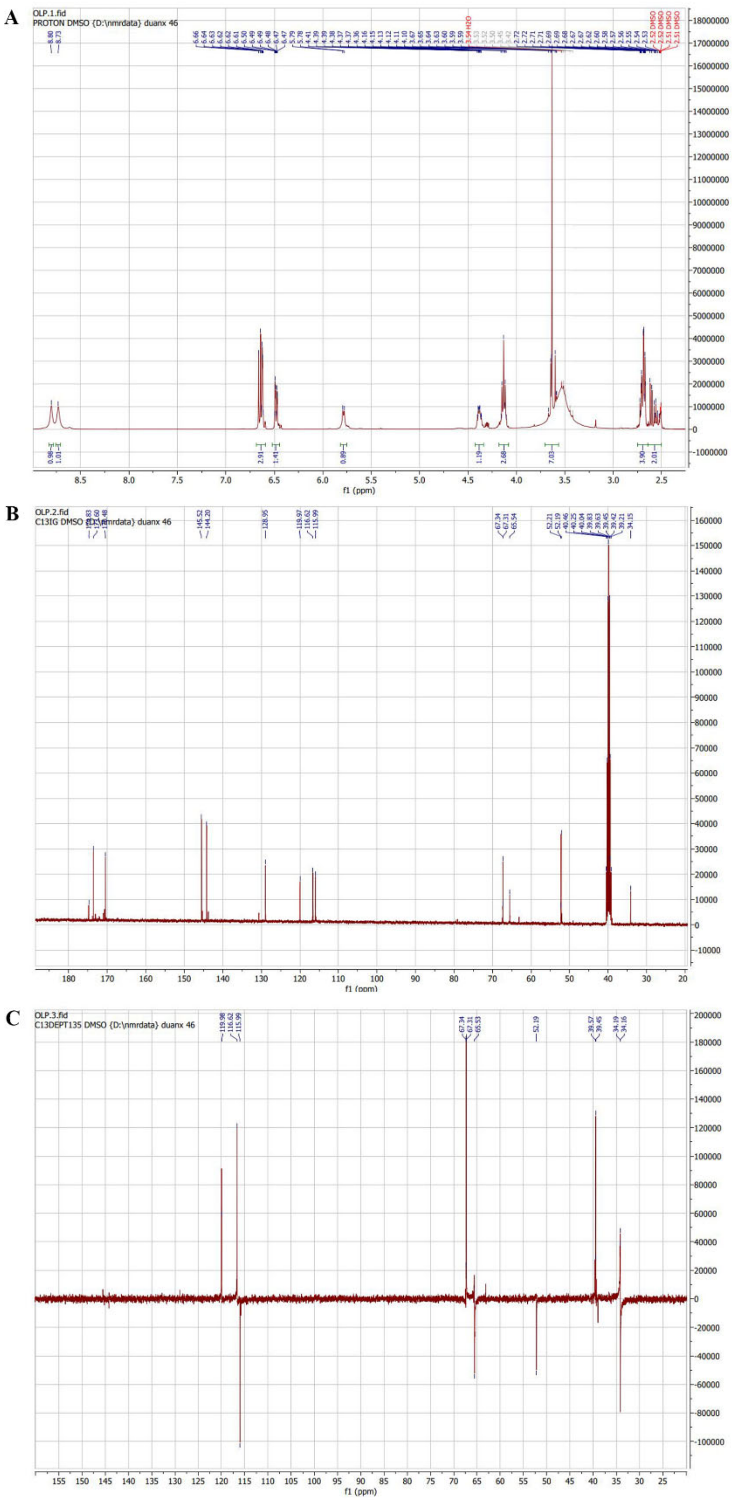

Figure S7. NMR spectra for the synthesised sample of OLP. OLP was dissolved in dDMSO and the NMR analysis was performed using the Bruker 400MHz Avance equipped with iProbe. The results can be seen for the (A) ^{1}H, (B) ^{13}C, and (C) ^{13}C DEPT-135 experiments.

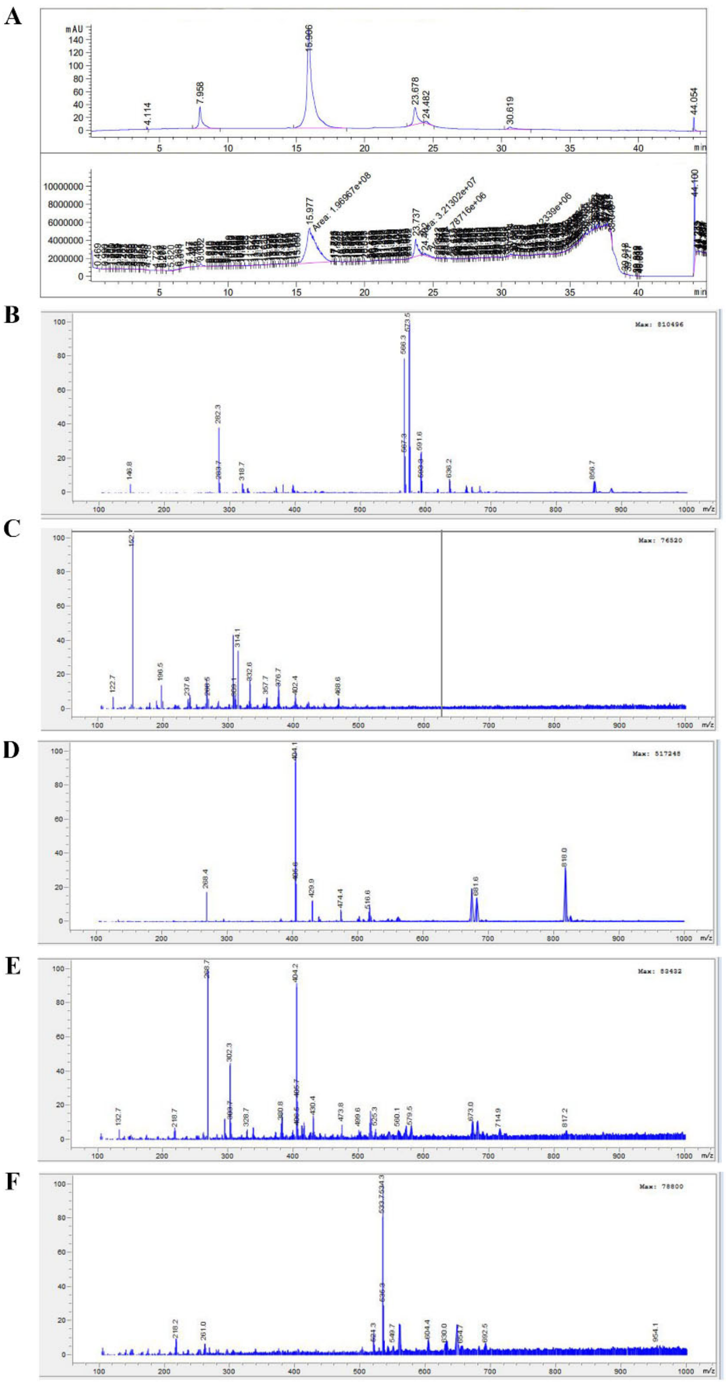

Figure S8. LC-MS analysis for the synthesised sample of OLP. (A) OLP was dissolved in DMSO and 5 µL was injected into a HPLC-DAD-ESI-MS system (Agilent 1260 Infinity II LC MSD, Australia). (B) The product ions for the major (15.98 min) and (C-F) minor peaks (8.00, 23.45, 24.40, and 30.61 min) can be seen. The major peak contains the OLP ion (m/z = 283, and 2m/z = 567).

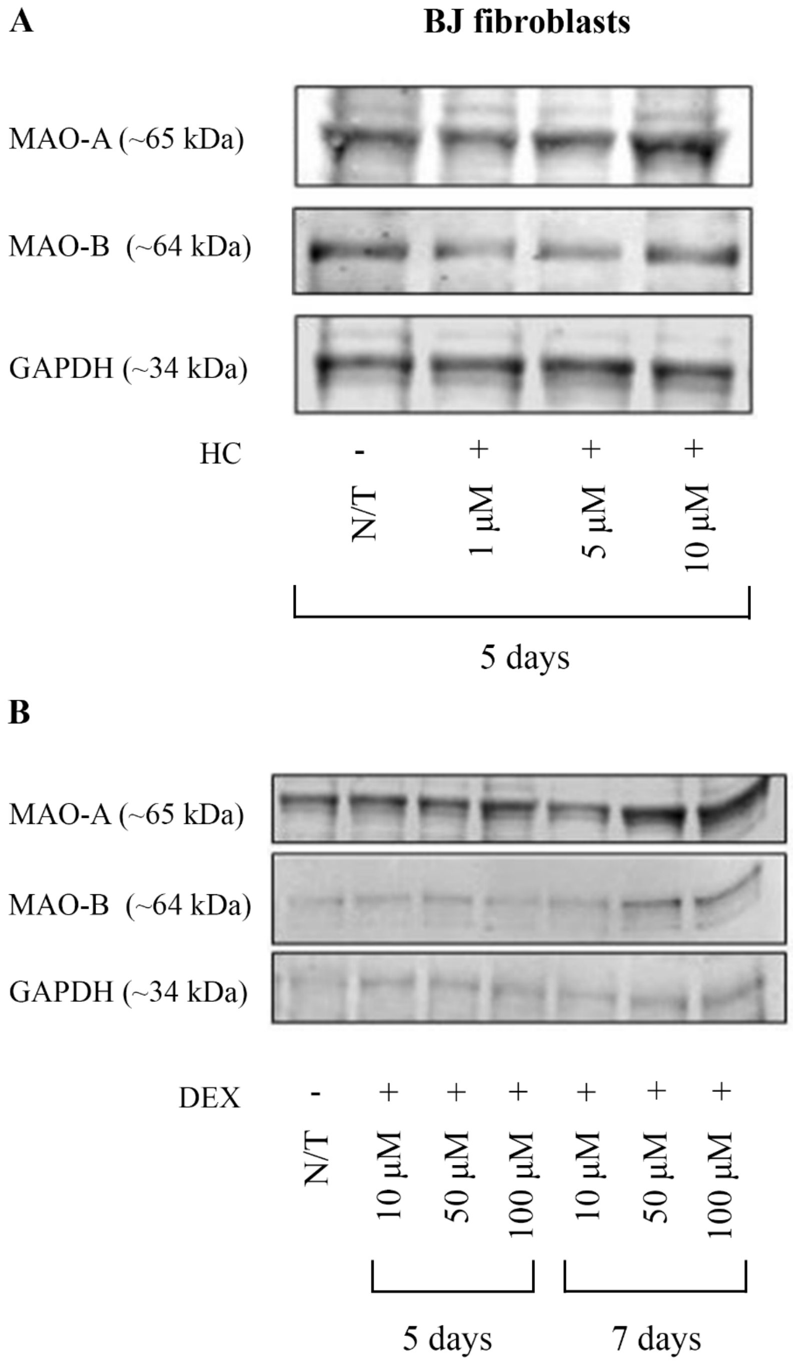

Figure S9. Upregulation of MAO protein expression by HC and DEX within BJ cells. (A) BJ cells were incubated with 1, 5, or 10 μM of HC for 5 days. (B) BJ cells were incubated with 10, 50, or 100 μM of DEX for a period of 5 or 7 days. Untreated (N/T) cells received normal growth medium. Western blot results are shown, where MAO-A/B expression was measured through immunodetection. "+" and "-" denotes the presence or absence of HC and DEX within cell culture groups, respectively.

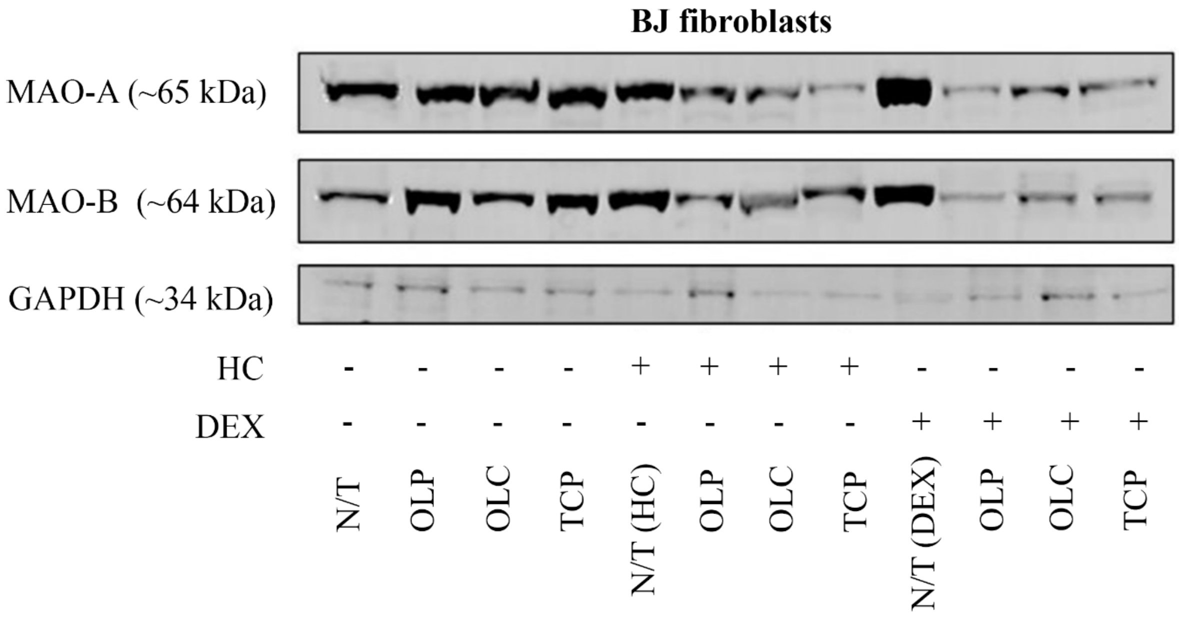

Figure S10. Potent inhibition of MAO enzyme expression by OLP and OLC within BJ cells. To stimulate MAO expression, BJ cells were incubated with 10 µM HC or 100 µM DEX. Following 5 or 7 days of incubation with HC or DEX, respectively, cells were washed and treated with 50 µM OLP, 50 µM OLC, or 5 µM TCP. Untreated cells received normal growth medium. Western blot results are shown, where MAO-A/B expression was measured through immunodetection. "+" and "-" denotes the presence or absence of HC and DEX within cell culture groups, respectively.
