# Supplementary material for: Identification and Evaluation of Olive Phenolics in the Context of Amine Oxidase Enzyme Inhibition and Depression: In Silico Modelling and In Vitro Validation
Source: Molecules. 2024 May 23;29(11):2446. doi: 10.3390/molecules29112446 (PMC11173677; doi:10.3390/molecules29112446)
Supplement: Supplementary file 1 [file molecules-29-02446-s001.zip › molecules-3018314-final.zip!/molecules-3018314.pdf]

molecules

MDPI

Article

Identification and Evaluation of Olive Phenolics in the Context of Amine Oxidase Enzyme Inhibition and Depression: In Silico Modelling and In Vitro Validation

Tom C. Karagiannis [1,2,3,4,*], Katherine Ververis [2,3], Julia J. Liang [1,2,5], Eleni Pitsillou [2,5], Siyao Liu [6], Sarah M. Bresnehan [2], Vivian Xu [2], Stevano J. Wijoyo [2,7], Xiaofei Duan [8], Ken Ng [6], Andrew Hung [5], Erik Goebel [9] and Assam El-Osta [1,7,10,11,12,13]

[1] Epigenetics in Human Health and Disease Program, Baker Heart and Diabetes Institute, 75 Commercial Road, Prahran, VIC 3004, Australia
[2] Epigenomic Medicine Laboratory at prospED Polytechnic, Carlton, VIC 3053, Australia
[3] Department of Clinical Pathology, The University of Melbourne, Parkville, VIC 3010, Australia
[4] Department of Microbiology and Immunology, The University of Melbourne, Parkville, VIC 3010, Australia
[5] School of Science, STEM College, RMIT University, Melbourne, VIC 3001, Australia
[6] School of Agriculture, Food and Ecosystem Sciences, Faculty of Science, The University of Melbourne, Parkville, VIC 3010, Australia
[7] Department of Diabetes, Central Clinical School, Monash University, Melbourne, VIC 3004, Australia
[8] Melbourne TrACEES Platform, School of Chemistry, Faculty of Science, The University of Melbourne, Parkville, VIC 3010, Australia
[9] Occhem Labs, LLC, 3510 Hopkins Place North, Oakdale, MN 55128, USA
[10] Department of Medicine and Therapeutics, The Chinese University of Hong Kong, Sha Tin, Hong Kong SAR, China
[11] Hong Kong Institute of Diabetes and Obesity, Prince of Wales Hospital, The Chinese University of Hong Kong, 3/F Lui Che Woo Clinical Sciences Building, 30-32 Ngan Shing Street, Sha Tin, Hong Kong SAR, China
[12] Li Ka Shing Institute of Health Sciences, The Chinese University of Hong Kong, Sha Tin, Hong Kong SAR, China
[13] Biomedical Laboratory Science, Department of Technology, Faculty of Health, University College Copenhagen, 1799 Copenhagen V, Denmark
* Correspondence: karat@unimelb.edu.au; Tel.: +61-38532-1290; Fax: +61-38532-1100

check for updates

Citation: Karagiannis, T.C.; Ververis, K.; Liang, J.J.; Pitsillou, E.; Liu, S.; Bresnehan, S.M.; Xu, V.; Wijoyo, S.J.; Duan, X.; Ng, K.; et al. Identification and Evaluation of Olive Phenolics in the Context of Amine Oxidase Enzyme Inhibition and Depression: In Silico Modelling and In Vitro Validation. *Molecules* **2024**, *29*, 0. https://doi.org/

Academic Editors: Ana Borota and Simona Funar-Timofei

Received: 3 May 2024
Revised: 15 May 2024
Accepted: 16 May 2024
Published: 21 May 2024

Copyright: © 2024 by the authors. Licensee MDPI, Basel, Switzerland. This article is an open access article distributed under the terms and conditions of the Creative Commons Attribution (CC BY) license (https://creativecommons.org/licenses/by/4.0/).

Abstract: The Mediterranean diet well known for its beneficial health effects, including mood enhancement, is characterised by the relatively high consumption of extra virgin olive oil (EVOO), which is rich in bioactive phenolic compounds. Over 200 phenolic compounds have been associated with *Olea europaea*, and of these, only a relatively small fraction have been characterised. Utilising the OliveNet^{TM} library, phenolic compounds were investigated as potential inhibitors of the epigenetic modifier lysine-specific demethylase 1 (LSD1). Furthermore, the compounds were screened for inhibition of the structurally similar monoamine oxidases (MAOs) which are directly implicated in the pathophysiology of depression. Molecular docking highlighted that olive phenolics interact with the active site of LSD1 and MAOs. Protein–peptide docking was also performed to evaluate the interaction of the histone H3 peptide with LSD1, in the presence of ligands bound to the substrate-binding cavity. To validate the in silico studies, the inhibitory activity of phenolic compounds was compared to the clinically approved inhibitor tranylcypromine. Our findings indicate that olive phenolics inhibit LSD1 and the MAOs in vitro. Using a cell culture model system with corticosteroid-stimulated human BJ fibroblast cells, the results demonstrate the attenuation of dexamethasone- and hydrocortisone-induced MAO activity by phenolic compounds. The findings were further corroborated using human embryonic stem cell (hESC)-derived neurons stimulated with all-trans retinoic acid. Overall, the results indicate the inhibition of flavin adenine dinucleotide (FAD)-dependent amine oxidases by olive phenolics. More generally, our findings further support at least a partial mechanism accounting for the antidepressant effects associated with EVOO and the Mediterranean diet.

Keywords: *Olea europaea*; olive phenolics; hydroxytyrosol; oleocanthal; oleohydroxypyretol; lysine-specific demethylase 1; monoamine oxidase

1. Introduction

Mental disorders accounted for 970.1 million cases of illness in 2019, with depressive disorders constituting the largest proportion of mental disorder disability-adjusted life years (37.3%) [1]. Depressive disorders were also reported to be more common in females than in males [1]. The symptoms and aetiology of major depressive disorder (MDD) are often heterogenous, which contributes to the complexity of accurately diagnosing and treating patients [2,3]. According to the Diagnostic and Statistical Manual of Mental Disorders (DSM-5) and the International Classification of Diseases (ICD-11), MDD is predominantly associated with a depressive mood or anhedonia [4,5]. These symptoms may be accompanied by other cognitive, behavioural, or neurovegetative changes that significantly impact an individual's ability to function [4,5].

The pathophysiological mechanisms underlying MDD require further elucidation. Formulated in the 1950s, the monoamine hypothesis suggests that depression is associated with a deficiency or imbalance of the monoamine neurotransmitters serotonin, norepinephrine, and dopamine [6,7]. Monoamine oxidases (MAO) catalyse the oxidative deamination of biogenic and xenobiotic amines in the central nervous system and peripheral tissues [8,9]. Although the MAO subtypes (MAO-A and MAO-B) share 70% sequence identity, the substrate and inhibitor specificities differ [9,10]. The monoamine hypothesis has also formed the foundation for the development of antidepressant drugs, which increase neurotransmitter levels. Several MAO inhibitors have been clinically approved for the treatment of depression, including tranylcypromine (TCP), selegiline, isocarboxazid, and phenelzine [11].

In addition to the monoamine hypothesis, the involvement of genetics, inflammation and oxidative stress, the hypothalamic–pituitary–adrenal axis, neurotrophins and neurogenesis, metabolism, and gut microbiota in the pathophysiology of MDD has been highlighted [7,12–14]. More recent studies have shown that epigenetic mechanisms, such as histone modifications and chromatin remodelling, play an important role in depression and the response to antidepressant treatments [15,16]. Interestingly, MAO-A and MAO-B are structurally related to lysine-specific histone demethylase 1 (LSD1) [17]. Through removal of methyl groups from mono- and dimethylated lysine 4 and lysine 9 residues on histone H3 (H3K4me1/2 and H3K9me1/2), gene expression is repressed or activated by LSD1, respectively [18]. Furthermore, LSD1 has been found to regulate the methylation status of other lysine residues in histone H3 and nonhistone proteins [18].

The MAO-A, MAO-B, and LSD1 enzymes require flavin adenine dinucleotide (FAD) as a co-factor [19]. TCP has been identified as a non-selective and irreversible inhibitor of the FAD-dependent amine oxidases [19]. Inhibition of the enzyme results in the formation of a covalent flavin adduct [19]. Due to the non-specific effects of irreversible inhibitors, the identification and development of reversible inhibitors has been of interest [20].

The Mediterranean diet, which is characterised by the high consumption of extra virgin olive oil (daily intake of 25–50 mL) (EVOO), has been associated with numerous health benefits [21,22]. Since the Seven Countries Study in the 1950s, which investigated the relationship between diet, lifestyle, and coronary heart disease, a number of epidemiological studies have demonstrated the association between the Mediterranean diet and a lower incidence of cardiovascular disease [21–25]. Moreover, adherence to the Mediterranean diet has been shown to have protective effects against depression and cognitive impairment [26]. In the SMILES trial conducted by Jacka et al., individuals with MDD were randomly assigned to a dietary support group and social support control group [27]. The dietary intervention involved supporting the consumption of 12 key food groups, including 3 tablespoons of olive oil per day [27]. At 12 weeks, 32.3% of the dietary support group

and 8.0% of the social support control group achieved remission criteria of a score of less than 10 on the Montgomery–Åsberg Depression Rating Scale [27]. Similarly, analysis of the Mod*i*MedDiet scores revealed that there was a significantly greater improvement in the dietary support at 12 weeks compared to the controls [27].

Following the discovery of the ibuprofen-like activity of the major olive polyphenol oleocanthal (OLC), considerable research efforts have focused on the phenolic fraction of EVOO, adding to the classical studies with the major fatty acid components, especially oleic acid [28]. It is now evident that bioactive olive phenolic compounds are key to the health effects of the Mediterranean diet. Over 200 hundred phenolic compounds have been linked to *Olea europaea*, and of these, only approximately half are commercially available [29]. To date, only a relatively small selection of olive phenolic compounds, such as tyrosol, hydroxytyrosol (HT), hydroxytyrosol acetate (HTA), homovanillyl alcohol (HVA), oleuropein (OLE), and OLC, have been investigated in models of human disease (Figure 1). The biological activity, such as antioxidant and anti-inflammatory properties, of HT, HTA, HVA, OLE, and OLC have been reported [30–32]. Many other phenolic compounds, such as oleohydroxypyretol (OLP), have not yet been investigated (Figure 1) [33]. Although clinical studies consistently highlight the beneficial effects of the diet, the molecular mechanisms of action of EVOO in the context of depression remain largely unexplored [27,34,35].

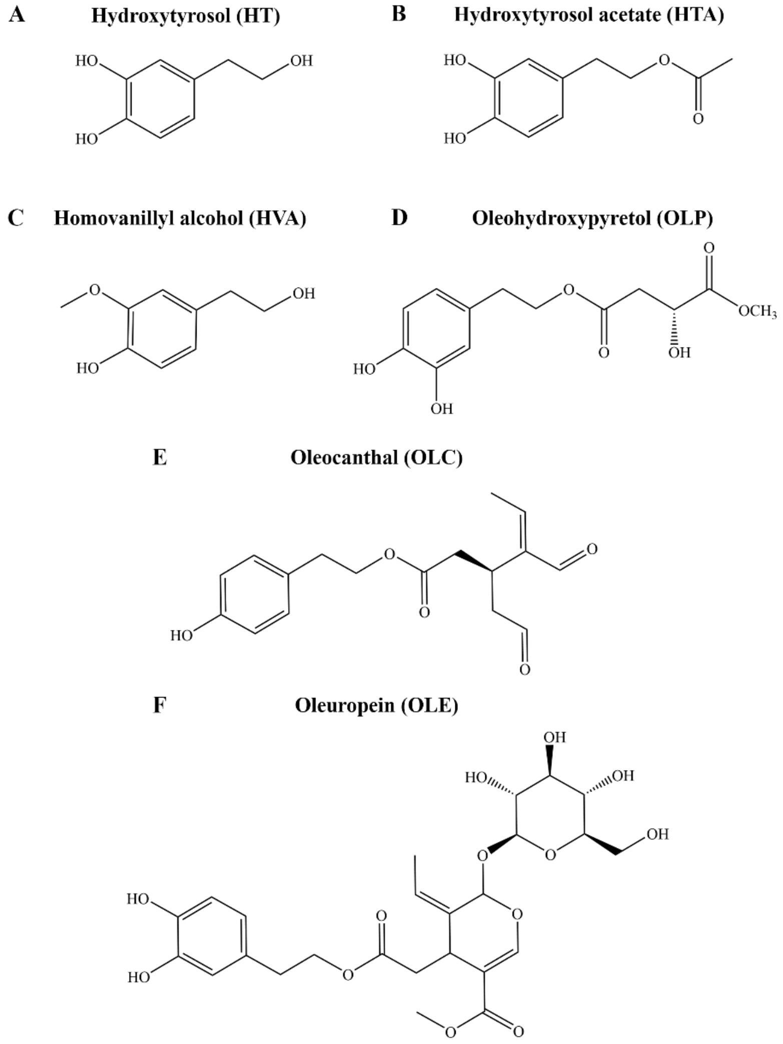

Figure 1. Chemical structures of key compounds from *Olea europaea*. The structures of the phenolic compounds (**A**) HT, (**B**) HTA, (**C**) HVA, (**D**) OLP, (**E**) OLC, and (**F**) OLE are shown.

In this study, the aim was to investigate HT, HTA, OLC, and OLP as potential inhibitors of FAD-dependent amine oxidases. In silico molecular modelling methods were initially used to examine the binding characteristics of the compounds against LSD1 and the MAO subtypes. The enzymatic inhibitory activity of the phenolic compounds in comparison to relevant control compounds, including TCP, was subsequently validated in vitro. Furthermore, the attenuation of the MAO-activating effects of dexamethasone (DEX), which is known to cause mood and cognitive changes, by the phenolic compounds was explored [36,37]. Normal human BJ skin fibroblasts, as well as human embryonic stem cell (hESC)-derived neurons, were utilised to highlight the biological effects of the olive phenolics.

2. Results and Discussion

2.1. In Silico Screen of Olea europaea Phenolics against FAD-Dependent Amine Oxidases

Following an initial screen of the OliveNet^{TM} library against LSD1 and the MAO subtypes, the binding characteristics of the phenolic compounds HT, HTA, OLC, and OLP were further investigated [38,39]. The OliveNet^{TM} library is a curated database consisting of 676 compounds derived from *Olea europaea* [29]. This includes 222 phenolic compounds that have been categorised into 13 subclasses [29].

2.1.1. Substrate-Binding Cavity of LSD1

Wu et al. previously identified 4-[5-(piperidin-4-ylmethoxy)-2-(*p*-tolyl)pyridine-3-yl]benzonitrile as a potent inhibitor of LSD1 [17]. The crystal structure of LSD1 in complex with the reversible inhibitor revealed that the 3-(piperidin-4-ylmethoxy)pyridine containing compound targets the substrate-binding cavity (Figure 2A) [40]. The pyridine ring is located in the middle of the substrate-binding site, while the piperidine ring resides in a negatively charged pocket formed by the side chains of N540 and D555 and the main-chain carbonyl groups of surrounding residues [40]. The 4-methylphenyl group occupies a relatively large hydrophobic pocket formed by residues V333, I356, F538, L677, L693, and W695 [40]. The 4-cyanophenyl group is positioned deep in the cavity, while the site of the central pyridine ring overlaps with the main-chain atoms of the methylated H3K4 peptide [40]. In this study, 4-[5-(piperidin-4-ylmethoxy)-2-(*p*-tolyl)pyridine-3-yl]benzonitrile was used as a positive control. The binding affinity of the reversible inhibitor for the substrate-binding cavity was predicted to be −9.5 kcal/mol (Figure 2A). The RMSD between the docked structure and co-crystallised ligand was 0.65 Å.

The top-ranking poses of the phenolic compounds generated from molecular docking study were evaluated. HT, HTA, OLC, and OLP were predicted to bind to the substrate-binding cavity with affinities of −5.3, −5.9, −7.1, and −7.2 kcal/mol, respectively (Figure 2B). The phenolic compounds were predicted to be positioned in the negatively charged pocket, similar to the piperidine ring of 4-[5-(piperidin-4-ylmethoxy)-2-(*p*-tolyl)pyridine-3-yl]benzonitrile. OLC and OLP were found to adopt a similar conformation within this region, as the residues within 5 Å of the ligands included T335, A539, N540, L547, W552, D555, F558, E559, F560, H564, Y761, S762, Y763, V764, Y773, N806, Y807, P808, A809, T810, and H812. In the study by Niwa et al., the piperidine ring of the reversible inhibitor was found to form a hydrogen bond with D555 [40]. Based on the molecular docking results, OLP and HT were predicted to form π–π stacking interactions with H812. HT also formed a hydrogen bond with the negatively charged residue D556.

The negatively charged amino acids at the entrance of the substrate cleft form the binding site for the N-terminal residues of histone H3 [41,42]. In order to investigate whether the bound phenolic compounds could disrupt the interaction between LSD1 and the histone H3 peptide, protein–peptide docking was performed. The results from the protein–peptide docking study revealed that the histone H3 peptide was preferentially binding to the substrate-binding pocket in the absence and presence of compounds (Figure 3).

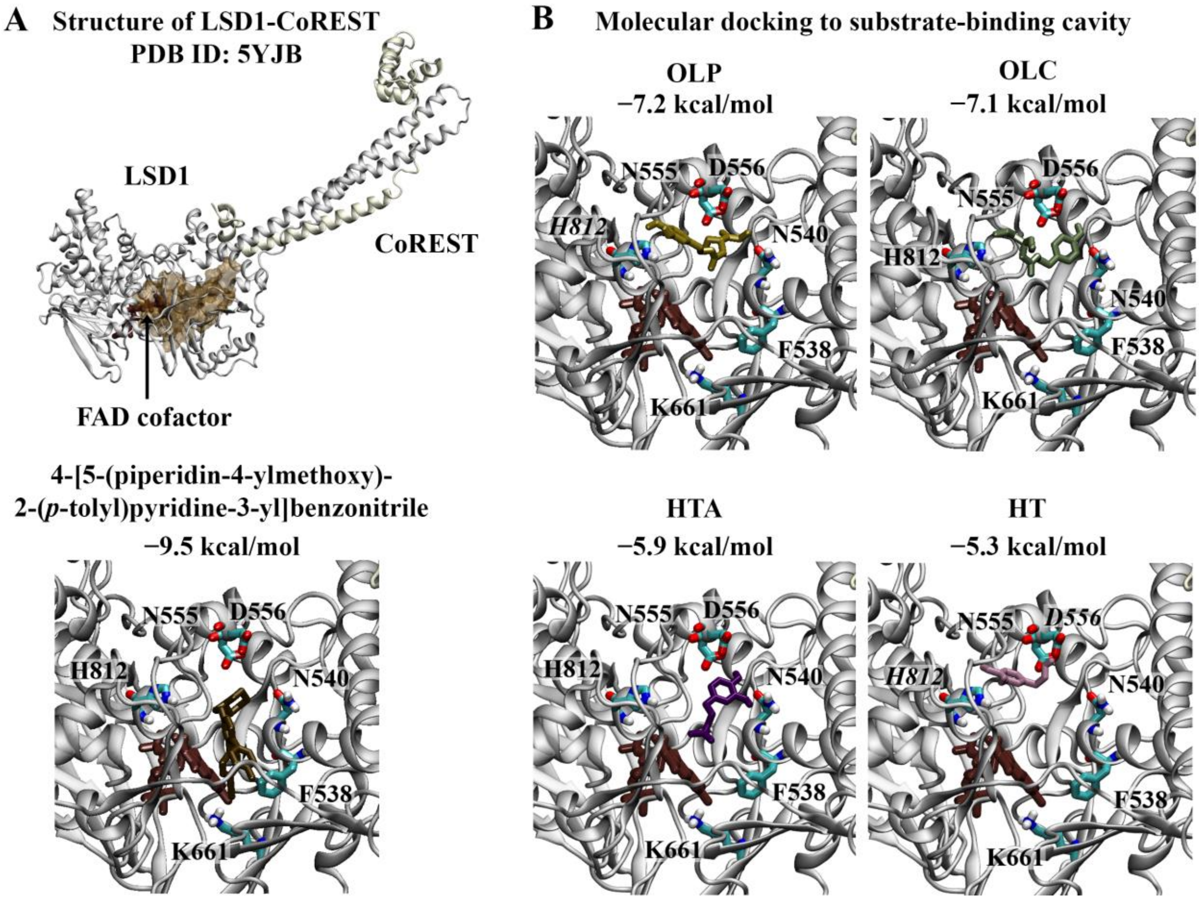

Figure 2. Molecular docking results for LSD1. (**A**) The crystal structure of LSD1 in complex with CoREST (PDB ID: 5YJB) is depicted. The FAD co-factor is labelled and predicted ligand-binding sites are coloured brown. 4-[5-(piperidin-4-ylmethoxy)-2-(*p*-tolyl)pyridin-3-yl]benzonitrile was used as the positive control inhibitor and was docked to the substrate-binding cavity of LSD1. The binding affinity was predicted to be −9.5 kcal/mol. (**B**) The phenolic compounds OLP, OLC, HTA, and HT were screened against the substrate-binding cavity and the binding affinities are provided (kcal/mol). Key residues are labelled, with those predicted to form hydrogen bonds and π–π stacking interactions italicised.

Redocking of the crystallographic histone H3 peptide was performed as a control. The top-ranking docking conformation compared to the crystal structure had an RMSD of 0.22 Å (Figure 3B). As reported by Forneris et al., the histone H3 peptide forms intermolecular contacts with LSD1 including salt bridges with D375, D553, D556, and E379 [43]. In accordance with the findings from the study by Forneris et al., R2 and R8 of the docked histone peptide formed salt bridges with D375, D553, D556, and E379. Several hydrogen bonds were also predicted to occur between the peptide and substrate-binding cavity of LSD1 (Table S1). The overall binding conformation of the histone H3 peptide in the presence of phenolic compounds in the substrate-binding cavity remained the same, with an RMSD of 1.0 Å compared to the crystallographic peptide (Figure 3C). In comparison to the crystal structure, fewer hydrogen bonds and salt bridges were predicted to form between R8 of the histone peptide and LSD1 (Table S1). The results suggest that there may be differences in the interactions between histone H3 and LSD1 when the phenolic compounds are bound to the substrate-binding cavity; however, further examination is warranted.

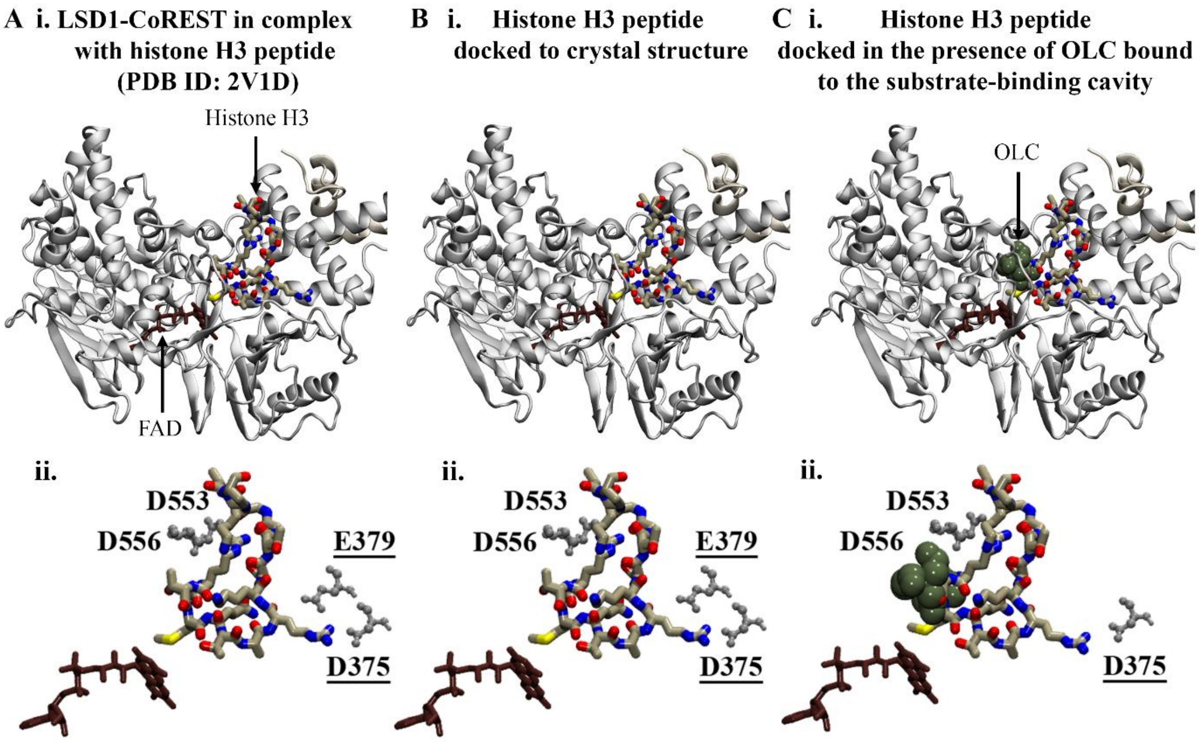

Figure 3. Protein–peptide docking results for histone H3 and LSD1. (**A**) Crystal structure of LSD1-CoREST in complex with the N-terminal residues of the histone H3 peptide. The FAD co-factor is coloured brown. Blind protein–peptide docking was performed to examine the preferential binding site of the histone H3 peptide in the presence of ligands bound to the substrate-binding cavity. The histone H3 peptide was docked to the crystal structure of LSD1-CoREST in the (**B**) absence and (**C**) presence of phenolic compounds bound to the substrate-binding cavity. The results are shown for OLC. (**Aii**–**Cii**) The protein–peptide interactions were evaluated using PDBePISA. The residues of the LSD1 substrate-binding cavity that were predicted to form salt bridges with R2 and R8 (underlined) of the histone H3 peptide are labelled.

2.1.2. Monoamine Oxidases

Crystal structures of the MAO-A monomer (Figure 4) and MAO-B dimer (Figure 5) in complex with harmine and safinamide, respectively, were selected for use [8,10]. Harmine is a reversible inhibitor that binds to the active center cavity of MAO-A, interacting with several key residues [8]. Harmine was predicted to bind to MAO-A with an affinity of −8.5 kcal/mol (Figure 4A). The RMSD between the docked structure and co-crystallised ligand was 0.40 Å. Based on the molecular docking results, harmine was predicted to form a π–π stacking interaction with the hydrophobic residue F208. Consistent with the structural study by Son et al., the amino acids Y69, N181, F208, V210, Q215, C323, I325, I335, L337, F352, Y407, and Y444 were found to be within 5 Å of the ligand [8]. Residues I335 and F208 in MAO-A, which correspond to Y326 and I199 of MAO-B, play an important role in the selectivity of reversible inhibitors [8]. OLP was predicted to bind with a similar affinity (−8.1 kcal/mol) to the positive control inhibitor, followed by HTA (−7.4 kcal/mol), OLC (−6.9 kcal/mol), and HT (−6.4 kcal/mol) (Figure 4B). OLC and OLP were predicted to form a π–π stacking interaction and hydrogen bond with F208 and Y444, respectively. HT and HTA formed hydrogen bonds with I180.

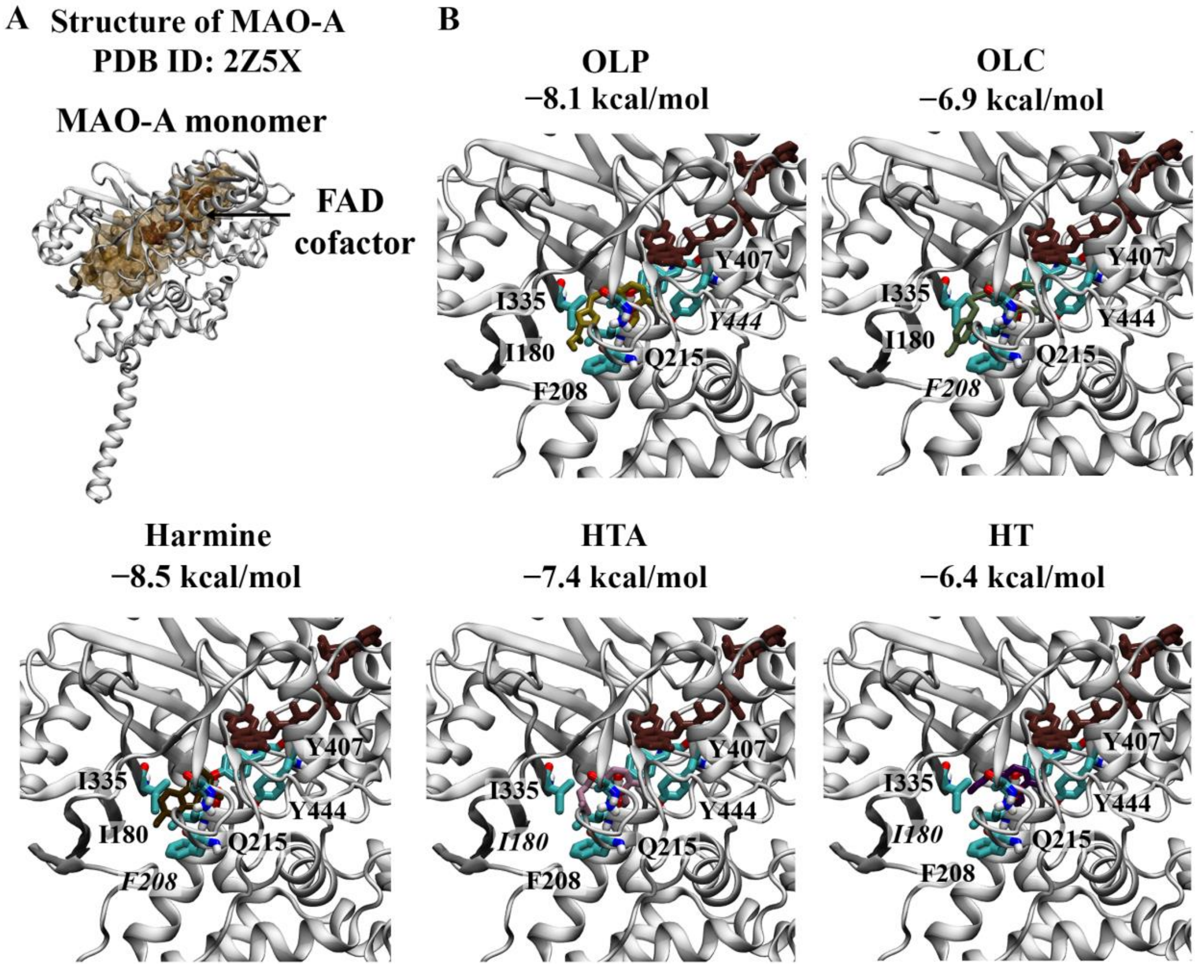

Figure 4. Molecular docking results for MAO-A. (**A**) The crystal structure of MAO-A (PDB ID: 2Z5X) is depicted. The FAD co-factor is labelled and predicted ligand-binding sites are coloured brown. Harmine was used as the positive control inhibitor and docked to the active site of MAO-A. The binding affinity was predicted to be −8.5 kcal/mol. (**B**) The phenolic compounds OLP, OLC, HTA, and HT were screened against the active site and the binding affinities are provided (kcal/mol). Key residues are labelled, with those predicted to form hydrogen bonds and π–π stacking interactions italicised.

The active site of MAO-B comprises the substrate and entrance cavities [10]. Safinamide is a reversible inhibitor that binds to MAO-B in an extended conformation, occupying both cavities [10]. Safinamide was predicted to bind to each subunit of the MAO-B dimer with an affinity of −9.9 kcal/mol and formed a π–π stacking interaction with the hydrophobic residue Y326 (Figure 5A). The RMSD values between the docked structure and co-crystallised ligand for chain A and chain B were 0.78 and 0.76 Å, respectively. Like the co-crystallised ligand, the docked structure of safinamide was surrounded by residues Y60, P102, F103, P104, W119, L164, L167, F168, L171, C172, I198, I199, Q206, I316, Y326, F343, Y398, and Y435 for each subunit.

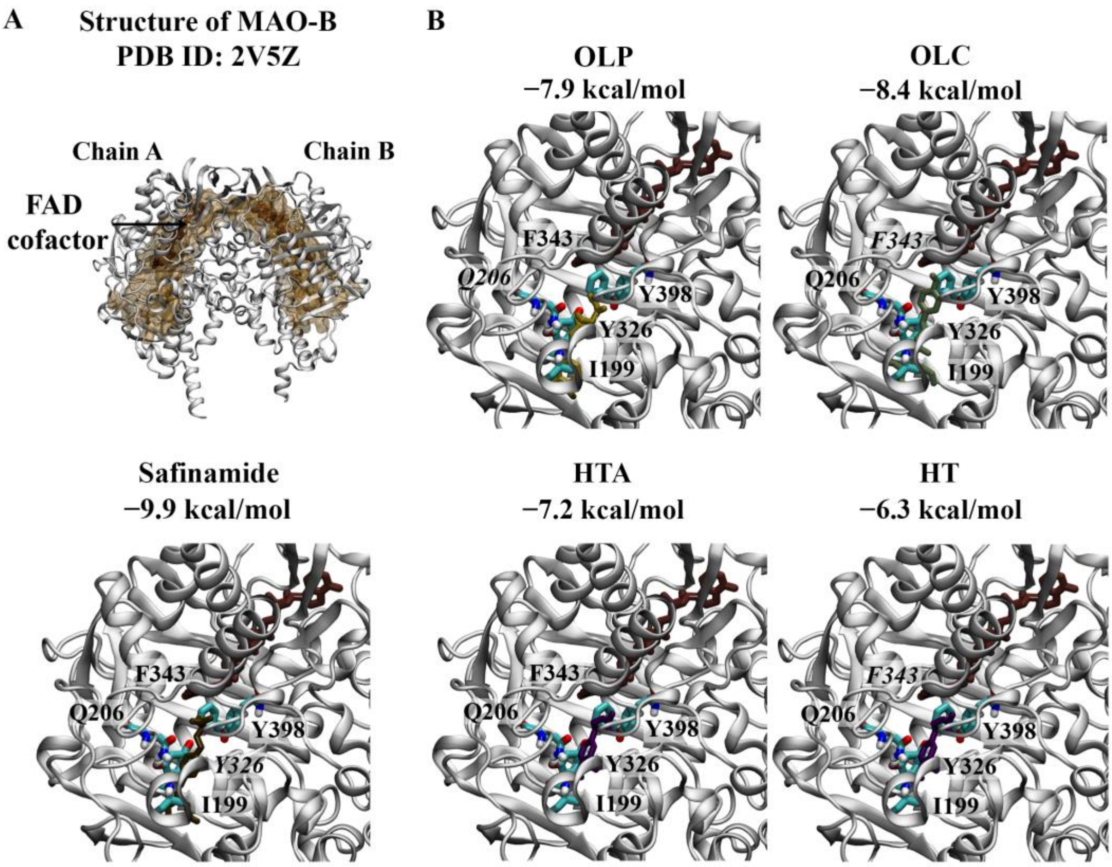

Figure 5. Molecular docking results for MAO-B. (**A**) The crystal structure of the MAO-B dimer (PDB ID: 2V5Z) is depicted. The FAD co-factor is labelled and predicted ligand-binding sites are coloured brown. Safinamide was used as the positive control inhibitor and docked to the active site of MAO-B. The binding affinity was predicted to be −9.9 kcal/mol. (**B**) The phenolic compounds OLP, OLC, HTA, and HT were screened against the active site, and the binding affinities are provided (kcal/mol). Key residues are labelled, with those predicted to form hydrogen bonds and π–π stacking interactions italicised. The results are shown for chain A of MAO-B.

As seen in Figure 5B, OLC (−8.4 kcal/mol) was predicted to have the strongest affinity for chain A of the MAO-B dimer, followed by OLP (−7.9 kcal/mol), HTA (−7.2 kcal/mol), and HT (−6.3 kcal/mol). OLP formed a hydrogen bond with Q206, while OLC and HT formed π–π stacking interactions with F343. A similar trend was observed for chain B of the MAO-B dimer: OLC (−8.3 kcal/mol), OLP (−7.9 kcal/mol), HTA (−7.1 kcal/mol), and HT (−6.3 kcal/mol). OLP was predicted to form hydrogen bonds with P102 and Y435. HT and HTA formed a π–π stacking interaction and hydrogen bond with F343 and I198, respectively.

2.2. *Viability of BJ Cells*

The activity of MAOs in fibroblasts has been previously found to remain relatively constant over 4–10 passages in young cell lines, with an increase (3–8 fold) being observed as the lines became senescent [44]. Due to the stable amine oxidase expression profile of BJ cells, the human foreskin-derived fibroblast cell line was selected for use in this study [44]. The viability of BJ cells was measured using the CellTiter-Blue Cell Viability Assay. The cells were treated with OLC or OLP at concentrations of 25, 50, 75, and 100 μM. Untreated BJ cells were used as controls. The BJ cells treated with OLP and OLC maintained their proliferation potential, similar to the untreated cells (Figure S1). González-Acedo et al. recently showed that the phenolic compounds HT and OLC significantly increased the

growth capacity of human skin fibroblasts in comparison to untreated controls at certain doses [45].

As seen in Figure S2, the relative viability of BJ cells decreased at the highest concentration of OLP (500 μM). A reduction in the viability of BJ cells treated with 500 μM OLC was also observed at each time point. At lower concentrations, up to 200 μM, the viability of BJ cells treated with OLC and OLP was largely maintained. Most notably, OLC and OLP were found to be non-toxic at the concentrations used in our further experiments. The IC_{50} value of OLC at 24 and 48 h was determined to be 123 and 111.3 μM, respectively. The IC_{50} value of OLP at 24 and 48 h was determined to be 219.5 μM and 257 μM, respectively.

2.3. Inhibition of LSD1 Demethylase Activity

The potential inhibitory activity of the phenolic compounds HT, HTA, OLC and OLP, was investigated through direct compound incubation with purified LSD1. TCP was used as the positive control. In comparison to TCP (IC_{50} = 110.5 μM), the phenolic compounds HT (IC_{50} = 0.039 μM), HTA (IC_{50} = 1.1 μM), and OLP (IC_{50} = 0.80 μM) were found to be more potent inhibitors of LSD1 (Figures 6A and S3). In a separate set of experiments (performed by Reaction Biology Corp.), the irreversible LSD1 inhibitor HCl 489479 was used as the positive control and had an IC_{50} value of 5.4 nM (Figure 6B). HTA (IC_{50} = 0.031 μM) exhibited a high level of inhibitory activity followed by OLP (IC_{50} = 0.12 μM), HT (IC_{50} = 0.57 μM), and OLC (IC_{50} = 12 μM) (Figures 6B and S3).

Furthermore, the BJ cells were incubated with normal growth medium or stimulated with DEX prior to treatment with TCP and the phenolic compounds. In cultures stimulated with DEX, a significant reduction in LSD1 activity was induced by TCP, HT, HTA, and OLP (Figure S4). Compared to the untreated cells, HT, HTA, and OLP increased the baseline levels of H3K4 mono-methylation in the BJ cells. The effects were more pronounced when the BJ cells were stimulated with DEX (Figure S4).

Cuyàs et al. initially employed a chemoinformatics approach to investigate the potential biomolecular targets of oleacein, a secoiridoid compound found in EVOO [46]. Various metabolic and epigenetic targets were identified, including LSD1 [46]. In a subsequent study, a combination of molecular docking, molecular dynamics (MD) simulations, and in vitro methods were used to further explore the mechanisms of action of oleacein against LSD1 [47]. The results from the in vitro assays demonstrated the ability of oleacein to act as an inhibitor of LSD1, as the mean IC_{50} value was found to be ~2.5 μmol/L [47].

In a study by Zheng et al., baicalin was characterised as the first flavonoid-based LSD1 inhibitor with an IC_{50} of 3.0 μM [48]. In addition to oleacein and baicalin, the inhibitory activity of natural polyphenols including resveratrol, curcumin, (-)-epigallocatechin gallate, and quercetin has been evaluated [49]. The IC_{50} values of resveratrol and curcumin have been reported to be 15 μM and 9.6 μM, respectively [49,50]. The chemical structures of resveratrol and curcumin have been used as scaffolds for the development of derivatives with potent inhibitory activities against LSD1 [50,51]. Capsaicin, which is a derivative of homovanillic acid, has also been found to inhibit LSD1 with an IC_{50} of 0.6 μM [52].

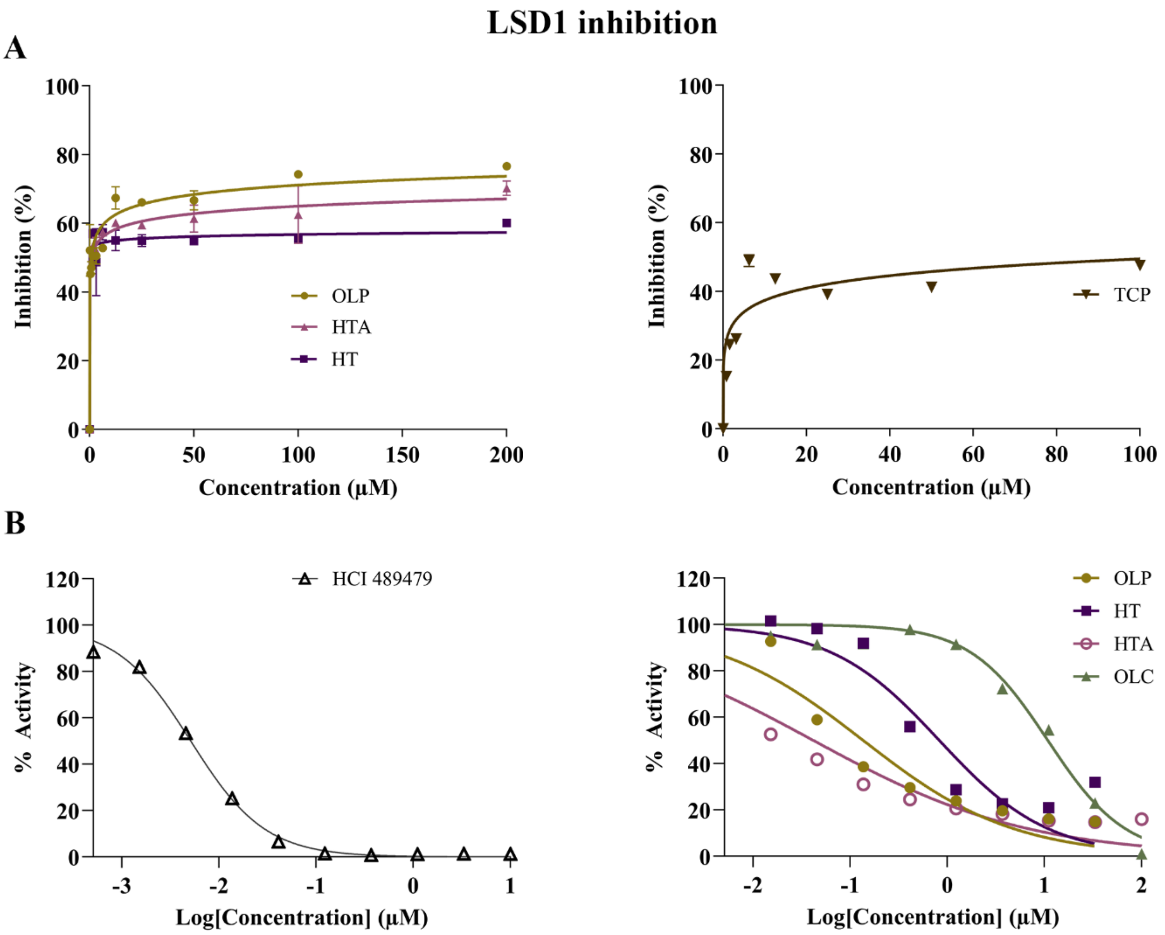

Figure 6. Inhibitory activity of phenolic compounds against LSD1. (**A**) The results of the control compound TCP and the phenolic compounds HT, HTA, and OLP from the direct enzymatic assays are provided. The data presented denote the mean ± SEM from duplicate (TCP, HTA, and OLP) and triplicate (HT) assays (representative results from *n* = 3 independent experiments). (**B**) A separate set of experiments were performed by Reaction Biology Corporation using the positive control inhibitor HCl 489479 and the phenolic compounds OLP, HT, HTA, and OLC. The demethylase activity (%) of LSD1 was measured for the control compound HCl 489479 and the phenolic compounds at concentrations ranging from 0–10 µM and 0–100 µM, respectively.

2.4. Inhibition of MAOs

The inhibitory activity of TCP, HT, HTA, OLC, and OLP was tested through direct compound screening with MAO-purified enzymes. The phenolic compound OLP (IC_{50} = 0.85 µM) was found to be a more potent inhibitor of MAO-A compared to OLC (IC_{50} = 1.9 µM) and the positive control TCP (IC_{50} = 1.4 µM). HT (IC_{50} = 0.43 µM) and HTA (IC_{50} = 0.70 µM) exhibited potent inhibition of MAO-A at a low micromolar range (Figure 7A). Moreover, HT, HTA, and OLP demonstrated greater inhibitory activity against MAO-A compared to MAO-B (OLP: IC_{50} = 32 µM, HT: IC_{50} = 4087 µM, HTA: IC_{50} = 91.42 µM) (Figure 7B). At a low micromolar range, TCP was found to have an IC_{50} of 0.80 µM for MAO-B.

The effects of DEX and hydrocortisone (HC) incubation on MAO activity in BJ cell cultures were examined (Figure 7C). DEX was shown to be a more effective stimulant of the MAO enzymes compared to HC. The results revealed that the phenolic compounds, OLC and OLP, were primarily able to downregulate MAO-A, MAO-B, and total MAO activity when basal levels were heightened with DEX (Figure 7C) or HC (Figure S5).

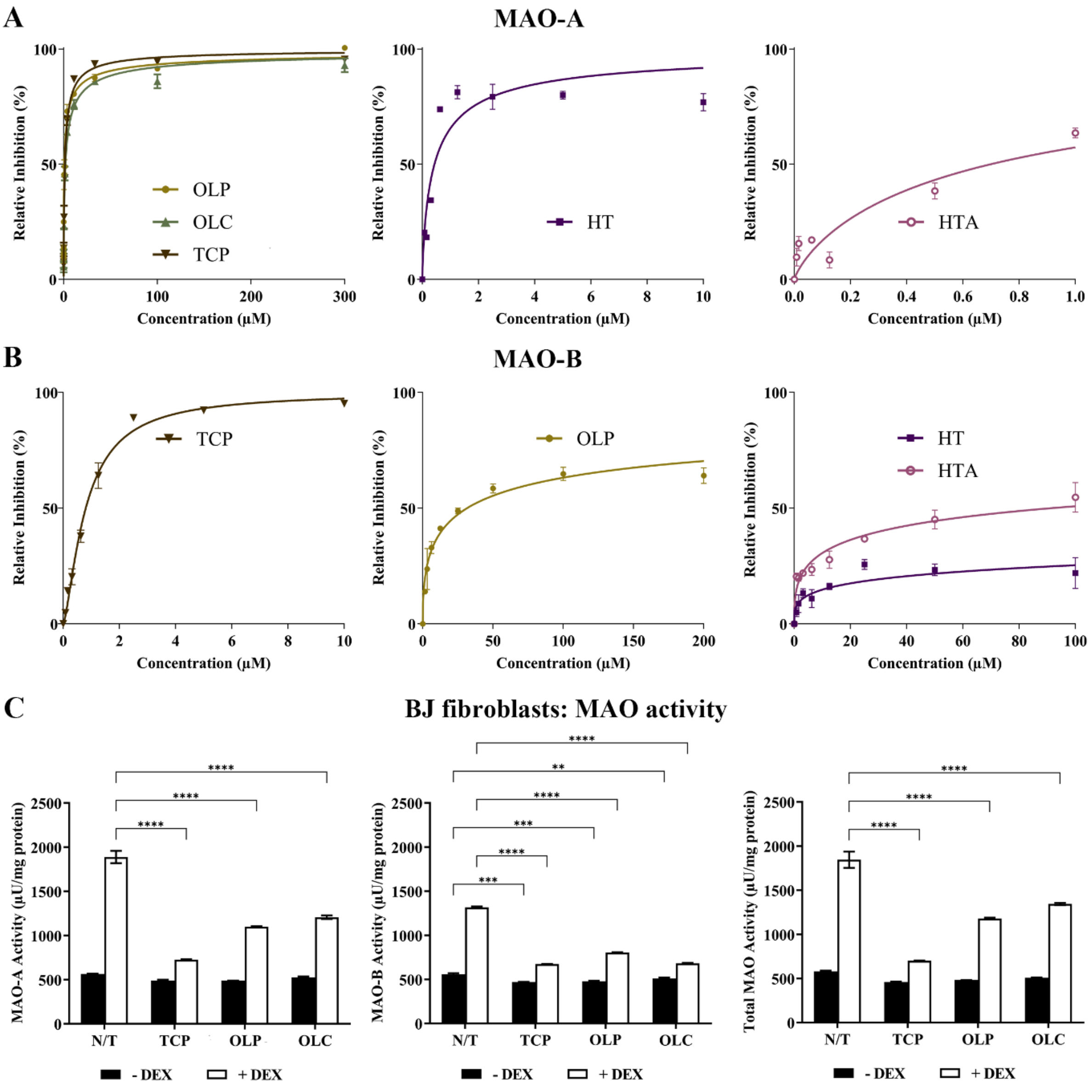

Figure 7. Potent inhibition of MAO-A by phenolic compounds. (**A**) Inhibition (%) of MAO-A by the control compound TCP and the phenolic compounds HT, HTA, OLP, and OLC. The data presented denote the mean ± SEM from duplicate (TCP, OLC, OLP, and HT) and triplicate (HTA) assays. (**B**) Inhibition (%) of MAO-B by the control compound TCP and the phenolic compounds HT, HTA, and OLP. The data presented denote the mean ± SEM from duplicate (TCP) and triplicate (HT, HTA, and OLP) assays. (**C**) The BJ cells were incubated with normal growth medium (−DEX) or 100 µM DEX (+DEX) for 7 days and treated with 50 µM OLP, 50 µM OLC, or 5 µM TCP for 24 h. The data obtained are represented as the mean ± SEM from duplicate assays. ** $p \leq 0.01$, *** $p \leq 0.001$, and **** $p \leq 0.0001$ quantified using a 2-way ANOVA with Tukey's post-hoc multiple comparisons test.

2.5. hESC-Derived Neurons: Inhibition of MAO Activity

Following the analysis of MAO activity within BJ fibroblast cells, hESC-derived neurons were selected as a novel and representative in vitro platform for modelling the MAO imbalance associated with depression (Figure S6). Over a 63-day period, cells derived from hESCs were incubated with growth factors to induce differentiation towards dorsal/ventral forebrain cortical neurons. At 14 d + 5 d, fine branching became apparent between neuronal progenitor cells. At days > 14 d + 5 d, the cells appeared dense with clear discrepancies between the cell body and dendritic connections. At this point, cells that were clustered

near neurospheres appeared shrunken and cells that were characteristic of mature neurons appeared most abundant away from these locations. Observational recordings of neurons at each stage of differentiation enabled us to deduce the time point at which neurons were healthiest and thus formed the basis of our treatment timeline.

Neuronal cells were differentiated until 31 days (14 d + 17 d). They were then pre-incubated with all-trans retinoic acid (ATRA, 1 µM) for 24 h and treated with NBM medium, TCP (5 µM), OLC (50 µM), or OLP (50 µM) for 24 h. Following stimulation with ATRA, TCP, OLC, and OLP significantly reduced MAO-A, MAO-B, and total MAO activity (Figure 8).

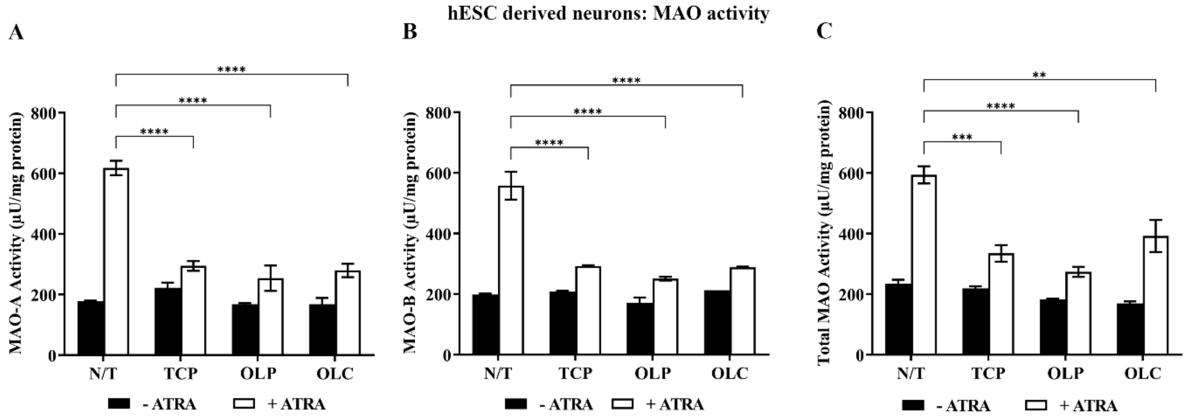

Figure 8. Inhibition of MAO activity by phenolic compounds within hESC-derived neurons stimulated with ATRA. hESC-derived neuron cultures were incubated with NBM medium or 1 µM ATRA for 24 h before treatment with 50 µM OLP, 50 µM OLC, or 5 µM TCP for a further 24 h. The total protein concentration was determined through a Bradford assay where the cells were then directly assayed. Through fluorometric kinetic detection, MAO-A (**A**), MAO-B (**B**), and the total MAO activity (**C**) were measured. The data obtained are represented as the mean ± SEM from duplicate assays. ** $p \leq 0.01$, *** $p \leq 0.001$, and **** $p \leq 0.0001$ quantified using a 2-way ANOVA with Tukey's post-hoc multiple comparisons test.

The in vitro inhibitory activity of phenolic compounds, particularly flavonoids, against MAOs has been reported [53]. Coumarins have been previously found to inhibit MAOs, with potent MAO-B inhibitory activity [53,54]. By using the structure of coumarin as a chemical scaffold, several derivatives have been synthesised and evaluated [10,54]. Binda et al. demonstrated that the inhibition constants of the coumarin analogs, 7-(3-chlorobenzyloxy)-4-(methylamino)methyl-coumarin and 7-(3-chlorobenzyloxy)-4-carboxaldehyde-coumarin, for MAO-B were 0.10 (MAO-A: 15.7) and 0.40 (MAO-A: 11.0) µM, respectively [10].

Harmine, which was used as a control compound in our molecular docking analysis, is a naturally occurring β-carboline alkaloid that exhibits high potency for MAO-A [8]. Like coumarin, the inhibition properties of β-carboline and carbazole derivatives have been investigated [55]. Furthermore, Zhang et al. investigated the selectivity of dietary phenolics for the inhibition of MAO-A and MAO-B [56]. Resveratrol (MAO-A: IC_{50} = 0.313 µM, MAO-B: IC_{50} = 15.8 µM) and isoeugenol (MAO-A: IC_{50} = 3.72 µM, MAO-B: IC_{50} = 102 µM) were found to be selective for MAO-A, while pterostilbene (MAO-A: IC_{50} = 13.4 µM, MAO-B: IC_{50} = 0.138 µM) was selective for MAO-B [56].

3. Materials and Methods

3.1. Molecular Docking to FAD-Dependent Monoamine Oxidases

3.1.1. Preparation of Protein Structures and Ligands

The structures of LSD1 in complex with CoREST (PDB ID: 5YJB), MAO-A monomer (PDB ID: 2Z5X), and MAO-B dimer (PDB ID: 2V5Z) were obtained from the RCSB Protein Data Bank [8,10,40]. Using PyMOL, the co-crystallised inhibitors and water molecules were removed from the protein structures, while the FAD co-factor was retained [57]. The

chemical structures of TCP, HT, HTA, and OLC were obtained from the National Center for Biotechnology Information PubChem database [58]. The structure of oleohydroxypyretol (OLP) was drawn using Chem3D 21.0.0 (PerkinElmer, Waltham, MA, USA).

The structures of LSD1-CoREST, MAO-A, and MAO-B were imported into AutoDockTools-1.5.7 and were prepared as macromolecules [59]. The 3D chemical structures of TCP, HT, HTA, OLC, and OLP were imported into PyRx and energy minimised using the universal force field through Open Babel (v.2.2.3) [60,61]. The energy-minimised structures and co-crystallised inhibitors were prepared as ligands using AutoDockTools-1.5.7 [59].

3.1.2. Ligand-Binding Site Analysis and Molecular Docking

The PrankWeb server was used to evaluate protein surfaces and predict potential ligand-binding sites (Table S2) [62]. The evolutionary conservation analysis option was selected [62]. Molecular docking was performed at an exhaustiveness of 2048 using AutoDock Vina [63]. The receptor grids (20 × 20 × 20 Å in size) were centered around the co-crystallised inhibitors 4-[5-(piperidin-4-ylmethoxy)-2-(*p*-tolyl)pyridin-3-yl]benzonitrile, harmine, and safinamide for LSD1, MAO-A, and MAO-B, respectively. The results were visualised using Maestro 13.2 and Visual Molecular Dynamics 1.9.3 [64,65]. Non-covalent protein–ligand interactions were evaluated using the default criteria for non-bonded interactions and were visualised using the Ligand Interaction Diagram Tool in Maestro 13.2 [64]. The in-place root mean square deviation (RMSD) values between the co-crystallised and docked ligands were calculated using PyMOL [57].

3.1.3. Protein–Peptide Docking

The crystal structure of LSD1-CoREST in complex with a histone H3 peptide was obtained from the RCSB PDB (ID: 2V1D) [43]. To study the potential effects of olive phenolic ligands on histone peptide binding, the crystallographic H3 peptide was removed from LSD1-CoREST, and the phenolic compounds were docked to the substrate-binding cavity using AutoDock Vina [63]. The docked phenolic compounds were retained in the substrate-binding cavity of LSD1 and protein–peptide docking was subsequently performed [66]. The HPEPDOCK 2.0 server was used to perform blind protein–peptide docking [66]. The structure of LSD1 without compounds bound to the substrate-binding cavity was utilised as a control. The top-ranking complexes generated from protein–peptide docking were uploaded to the Proteins, Interfaces, Structures, and Assemblies (PDBePISA) server to evaluate hydrogen bonds and salt bridges [67]. PDBePISA detects hydrogen bonds if the distance between the heavy atoms of the donor and acceptor is less than 3.89 Å [67]. When the hydrogen atom is present, the acceptor-H distance must be $\leq$4 Å and the angle A-H-D must be between 90 and 270 [67]. The distance for a salt bridge is 4 Å [67]. The in-place RMSD values between the co-crystallised and docked peptide were calculated using PyMOL [57].

3.2. Cell Culture

3.2.1. BJ Cells

Publicly available cell line cultures were purchased from the American Type Culture Collection (ATCC). All growth media and various culture reagents were heated to 37 °C prior to use. BJ human fibroblasts (ATCC CRL-2522) were cultured in T-75 flasks (Corning, 430641U, Corning, NY, USA) with 10 mL Dulbecco's modified Eagle medium/nutrient mixture F12 (Thermo Fisher Scientific, Gibco 11330032, Waltham, MA, USA) supplemented with 10% heat-inactivated fetal bovine serum (HyClone Characterized FBS, SH30084.02) and 1% penicillin–streptomycin (Thermo Fisher Scientific, Gibco 15140122, Waltham, MA, USA). The medium was renewed once or twice per week and subcultured at a 1:3 ratio once per week.

The cell culture medium was aspirated, and confluent flasks were washed twice with D-PBS (Thermo Fisher Scientific, Gibco 14190144, Waltham, MA, USA). The cells were detached using trypsin-EDTA (5 mL, 5 min, Thermo Fisher Scientific, Gibco R001100,

Waltham, MA, USA). Growth medium (7 mL) was then added to the flask followed by gentle aspiration to obtain a single cell suspension. The cell suspension was then transferred into a 15 mL tube (Corning, 430791, Corning, NY, USA) for 5 min centrifugation at 335 g. The resulting cell pellet was then resuspended in a fresh growth medium and aliquoted into new culture vessels at an appropriate subculturing ratio. The cell cultures were incubated at 37 °C with 5% CO_2 atmospheric content. The growth medium was removed from the T-75 flasks and washed twice with D-PBS. Fresh growth medium was added to the flasks, and using a cell scraper (Thermo Fisher Scientific, Thermo Scientific 179693, Waltham, MA, USA), the cells detached through mechanical disruption. The cell suspension was transferred to a 15 mL tube and centrifuged (350 g for 5 min). The samples were either stored at −80 °C or incubated on ice for subsequent procedures.

3.2.2. hESC-Derived Neurons

The hESC (H9) cell line was kindly provided by Dr Ana Antonic Baker from the Department of Neuroscience, Monash University. The cells were grown at 37 °C with 5% (*v*/*v*) CO_2 atmospheric content within a humidified cell culture incubator. To promote the differentiation of human pluripotent stem cells (hPSCs), growth factors and small molecules were mixed with neural basal cell culture medium (NBM) (Tables S3 and S4). The cells were driven into two weeks of neural induction where hPSCs were passaged via 0.5 mM ethylenediaminetetraacetic acid (EDTA) (Thermo Fisher Scientific, Invitrogen 15575020, Waltham, MA, USA) and plated onto laminin-coated plates (Thermo Fisher Scientific, Gibco A29248, Waltham, MA, USA) submersed in TeSR-E8 basal media (Thermo Fisher Scientific, Gibco A1517001, Waltham, MA, USA). After 24 h, the media were replaced with NBM in order to induce differentiation towards dorsal/ventral forebrain cortical neurons. Following initial neural induction, media containing cell colonies were supplemented with basic fibroblast growth factor (bFGF) for 7 days.

After 14 days, early neurospheres were harvested and transferred into a 96-well U-bottom ultralow attachment plate (Sigma-Aldrich, Corning CLS7007, St. Louis, MO, USA). For 21 days, early neurospheres were suspended in NBM with epidermal growth factor (EGF) and bFGF to promote neural precursor cell expansion. Neurospheres were then cultured for an additional 14 days, with NBM media replaced twice a week. Microplates of 24 wells were coated with phosphate-buffered saline (PBS) (1.8 mM HH_2PO_4, 2.7 mM KCl, 137 mM NaCl, and 10 mM Na_2HPO_4, *w*/*v*)-diluted laminin of 10 µg/mL concentration and left to incubate for 2 h. The laminin was then aspirated and 500 µL of NBM (warmed to 37 °C) was added. Neurospheres were seeded and manually dissociated to a single cell suspension by passing them through a sterile pipette tip and into laminin-coated wells. The NBM was renewed three times a week, replacing half the volume of each well with fresh media.

Following treatment of MAO stimulants and olive phenolics, the cells were washed twice with D-PBS (Thermo Fisher Scientific, Gibco 14190250, Waltham, MA, USA). Using 200 µL of Trypsin-EDTA solution (Thermo Fisher Scientific, Gibco R001100, Waltham, MA, USA), the cells detached from the wells (2 min exposure) and 300 µL of medium was added to neutralise trypsin activity. The cell suspension solution was transferred to a 15 mL tube (Corning, 430791, Corning, NY, USA) and centrifuged for 5 min at 350× *g*. The samples were either stored at −80 °C or incubated on ice for subsequent procedures.

3.3. Cell Treatments and Analyses

3.3.1. Olea Europaea Phenolic Compounds

The purity of OLC was ≥95% (Sigma-Aldrich SMB00810, St. Louis, MO, USA), that of HT was ≥ 98% (Cayman Chemicals 70604, Ann Arbor, MI, USA), and that of HTA was ≥98% (Enzo Life Sciences, ALX-350-404-M050, Farmingdale, NY, USA). OLP, which was previously identified as a novel phenolic compound [33], was synthesised by Occhem Labs, LLC (~90%; relevant LC-MS and NMR spectra in Supplementary Methods,

Figures S7 and S8). TCP (Sigma-Aldrich, Calbiochem 616431, St. Louis, MO, USA) was used as a positive control inhibitor for LSD1 and the MAOs (≥97%).

3.3.2. Western Blotting

Western blots were performed on BJ cells and hESC-derived neurons to analyse H3K4 methylation status and MAO-A/B expression, respectively. Detailed instructions are outlined in the Supplementary Methods and Tables S5 and S6.

3.3.3. Cell Viability

The BJ cells were seeded at densities of 10,000 cells in black flat bottom 96-well plates (Nalge Nunc, Penfield, Rochester, NY, USA) and treated with a dose–response of OLC (0–500 μM) and OLP (0–500 μM) for 24 h, 48 h, 72 h, and 7 days at 37 °C, 5% (*v*/*v*) CO_2. Cell viability was measured using the Cell-Titer Blue® Assay kit (Promega, Madison, WI, USA) according to the manufacturer's instructions. Cell-Titer Blue reagent was added to each well and incubated for 4 h at 37 °C with 5% (*v*/*v*) CO_2 before the fluorescence intensity was read using the CLARIOstar Microplate Reader (BMG Labtech, Ortenberg, Germany).

A Nikon Eclipse Ts2 light microscope (Nikon Ti TS2-S-SM, Tokyo, Japan) was used to image untreated and treated BJ cells (OLP or OLC: 0–100 μM) at 4× objective magnification at 20, 44, and 120 h (Figures S1 and S2). Moreover, the IC_{50} values of OLC and OLP at 24 and 48 h were measured.

3.3.4. LSD1 Activity and H3K4 Mono-Methylation in BJ Cells

The BJ cell cultures were incubated with normal growth medium or stimulated with DEX (50 μM) for 72 h prior to treatment with TCP (5 μM), OLP (50 μM), HT (50 μM), or HTA (50 μM) for 48 h. As described in the Supplementary Information, nuclear proteins extracted from the BJ cultures were directly assayed for LSD1 activity (Epigentek P-3076, Farmingdale, NY, USA). Fluorescence was read on a CLARIOstar Microplate Reader (BMG Labtech, Ortenberg, Germany) at Ex/Em = 530/590 nm (Figure S4). Histone proteins were extracted from the treated cultures and were analysed through Western blotting. The mono-methylation status of lysine on histone 3 (H3K4) was determined via immunodetection (Table S6).

3.3.5. MAO Upregulation in BJ Cells

The BJ cells were initially treated with varying time and dose concentrations of hydrocortisone (HC) and DEX to optimise the cell treatments (Figures S9 and S10). The cells were incubated with 1, 5, or 10 μM of HC for 5 days. The BJ cells were also incubated with 10, 50, or 100 μM of DEX for 5 or 7 days. Untreated cells received normal growth medium. The cells were collected, homogenised, and analysed through Western blotting where MAO-A/B expression was determined through immunodetection.

The BJ cells were subsequently incubated with 10 μM HC or 100 μM DEX to stimulate MAO expression. Following 5 or 7 days of incubation, for HC or DEX, respectively, the cells were washed and treated with 5 μM TCP, 50 μM OLC, or 50 μM OLP. The untreated BJ cells received normal growth medium. The cells were collected, homogenised, and analysed through Western blotting where the expression of MAO-A/B was determined through immunodetection of protein subtypes.

3.3.6. MAO Upregulation in hESC-Derived Neurons

An in vitro model of MDD was designed for stem cell-derived neuronal cells through incubation of 1 μM all-trans retinoic acid (Sigma-Aldrich PHR1187, St. Louis, MO, USA). All cell treatments were conducted in duplicate and replicated using independently differentiating neuronal plates. At 31 days of incubation, the cells were incubated with normal NBM medium or 1 μM all-trans retinoic acid (ATRA) for 24 h, followed by treatment with either 5 μM TCP, 50 μM OLC, or 50 μM OLP for a further 24 h.

3.3.7. MAO-A/B and Total MAO Activity Assay

Activity assays were commercially sourced from BioVision (Abcam, BioVision ab241031, Waltham, MA, USA) and were performed according to manufacturer's instructions. The treatment groups were plated in duplicate on a black, flat 96-well microplate. The cells were homogenised in assay buffer using a microtube mixer at 4 °C and centrifuged at 10,000× *g* for 10 min. A Bradford assay was performed to determine the total protein concentration within each cell pellet (200 µg/well for BJ cells, 40 µg/well for hESC-derived neurons). Approximately 40 µL of supernatant was loaded into wells along with 10 µL of either selegiline for MAO-A activity, clorgyline for MAO-B activity, or assay buffer for total MAO activity. A H_2O_2 standard was pipetted as supplied and 50 µL of reaction mix was pipetted into all wells. Using a CLARIOstar Plus Microplate Reader, (BMG Labtech, Ortenberg, Germany), fluorescence was measured kinetically at 25 °C for 60 min with Ex/Em = 535/587 nm.

3.4. Inhibitor Screening Assays

3.4.1. LSD1 Direct Screening Assay

The inhibitory activity of TCP, HT, HTA, and OLP against purified LSD1 was measured using a commercially available histone demethylase LSD1 inhibitory screening assay core kit (Epigentek P-3075A, Farmingdale, NY, USA) according to the manufacturer's instructions. TCP was used as a positive control inhibitor. The inhibitory activity of TCP was tested in 8-dose IC_{50} mode with 2-fold serial dilution starting at 100 µM, while the inhibitory activity of HT and HTA was tested in 8-dose IC_{50} mode with 2-fold serial dilution starting at 200 µM. The inhibitory activity of OLP was tested in 11-dose IC_{50} mode with 2-fold serial dilution starting at 200 µM. Fluorescence was measured using a CLARIOstar Plus Microplate Reader (BMG Labtech, Ortenberg, Germany) at excitation and emission wavelengths of 530 nm and 590 nm, respectively (Ex/Em = 530/590 nm).

The inhibitory activity of HCl 489479, OLC, and OLP was measured using a fluorescence coupling enzyme assay (performed by Reaction Biology Corporation). The inhibitory activity of HT, HTA, OLC, and OLP was tested in 10-dose IC_{50} mode with 3-fold serial dilution in duplicate starting at 100 µM. HCl 489479 was used as a positive control, with its inhibitory activity being tested in 10-dose IC_{50} mode with 3-fold serial dilution in duplicate starting at 10 µM. All compounds were pre-incubated for 30 min with the enzyme before the addition of 10 µM histone H3(1-21)K4me2 peptide substrate to start the reaction. Fluorescence was measured using an EnVision Multimode Plate Reader at Ex/Em = 535/590.

3.4.2. MAO-A/B Direct Screening Assay

TCP, HT, HTA, OLC, and OLP were screened for their inhibitory activity against purified MAO-A (Sigma-Aldrich MAK295, St. Louis, MO, USA) and MAO-B (Sigma-Aldrich MAK296, St. Louis, MO, USA) using commercially available inhibitor screening kits. TCP was used as a positive control. The assays were conducted for MAO-A and MAO-B according to the manufacturer's instructions. The inhibitory activity of TCP, OLC, and OLP against MAO-A was tested in 11-dose IC_{50} mode with 3-fold serial dilution starting at 300 µM. The inhibitory activity of HT and HTA was tested against MAO-A in 8-dose IC50 mode with 2-fold serial dilution starting at 10 µM and 1000 nM, respectively. The inhibitory activity of TCP and OLP was tested against MAO-B in 8-dose IC_{50} mode with 2-fold serial dilution starting at 10 and 200 µM, respectively. The inhibitory activity of HT and HTA was tested in 8-dose IC_{50} mode with 2-fold serial dilution starting at 100 µM. Using a CLARIOstar Plus Microplate Reader (BMG Labtech, Ortenberg, Germany), fluorescence was measured kinetically for 30 min at 25 °C (MAO-A) or 60 min at 37 °C (MAO-B) at Ex/Em = 535/597 nm.

3.5. Statistical Analyses

Statistical analyses were performed using GraphPad Prism 9.5.1. (GraphPad Software, San Diego, CA, USA). For comparisons involving treatment groups compared to the controls, a two-way analysis of variance (ANOVA) was conducted, followed by Tukey's post hoc multiple comparisons test to determine statistical significance. The data presented are shown as the mean ± standard error of the mean (SEM): * $p < 0.05$, ** $p < 0.01$, *** $p < 0.001$, and **** $p < 0.0001$. A non-regression analysis was performed to determine IC_{50} values.

4. Conclusions

Overall, the bioactive properties of olive phenolic compounds including HT, HTA, OLC, and OLP were investigated. The molecular modelling results show that the phenolic compounds target the substrate-binding cavity of LSD1 and the active site of the structurally similar MAO-A and MAO-B subtypes. Based on our in vitro experiments, the compounds were found to exhibit inhibitory activity against the epigenome-modifying LSD1 and MAOs. Modulation of MAO-A and MAO-B by these phenolic compounds is encouraging in the context of further development for potential use in depression. Given the potential of clinically approved antidepressant drugs and phenolic compounds to inhibit both LSD1 and the MAOs, the role of epigenetic regulation of small molecule inhibitors requires further investigation. More generally, our findings highlight the molecular mechanisms of action of olive phenolics in models of disease and represent the initial steps in delineating the molecular mechanisms associated with the well-known health benefits of the Mediterranean diet. As stated earlier, there are over 200 phenolic compounds associated with *Olea europaea* and many remain uncharacterised. Therefore, further synthesis of olive phenolic compounds and thorough characterisation of bioactivity is an important future direction in the field.

Supplementary Materials: The following supporting information can be downloaded at: https://www.mdpi.com/article/10.3390/molecules29110000/s1. Figure S1. Assessment of cell viability using the CellTiter-Blue® Assay kit. The BJ cells were either untreated (N/T) or treated with increasing concentrations (25, 50, 75, and 100 μM) of OLC and OLP. The BJ cells were imaged at 4× objective magnification at 20, 44, and 120 h using a Nikon Eclipse Ts2 light microscope. Figure S2. Viability of BJ cells. (A) The relative cell viability (%) of BJ cells treated with 0–500 μM OLC and OLP was measured at 24 h, 48 h, 72 h, and 7 days. The results are shown for 50, 100, 150, 200, and 500 μM. The error bars represent the % SEM from duplicate assays. The IC_{50} values were calculated at 24 h and 48 h. The data presented denote the mean ± SEM from duplicate assays (representative results from $n = 3$ independent experiments). Figure S3. Potent inhibition of LSD1 by phenolic compounds. (A) Inhibition (%) of LSD1 by the control compound TCP and the phenolic compounds HT, HTA, and OLP at concentrations of 12.5, 25, 50, and 100 μM. The data presented denote the mean ± SEM from duplicate (TCP, HTA, and OLP) and triplicate (HT) assays (representative results from $n = 3$ independent experiments). A nonlinear regression analysis was performed to determine the IC_{50} values: TCP = 110.5 μM, HT = 0.039 μM, HTA = 1.1 μM, and OLP = 0.80 μM. (B) LSD1 was preincubated with the control compound HCl 489479, OLP, and OLC prior to the addition of the peptide substrate. The demethylase activity (%) of LSD1 was measured for the control compound HCl 489479 and the phenolic compounds (OLP and OLC) at concentrations ranging from 0–10 μM and 0–100 μM, respectively. The IC_{50} values were determined to be 5.4 nM, 12 μM, and 0.12 μM for HCl 489479, OLC, and OLP, respectively (as performed by Reaction Biology Corporation, fluorescence coupling enzyme assay). Figure S4. Phenolic compounds reduce LSD1 activity in BJ fibroblasts stimulated with DEX. (A) The BJ cell cultures were stimulated with DEX (50 μM) or incubated with normal growth medium for 72 h prior to treatment with the established inhibitor TCP (5 μM) or the phenolic compounds OLP (50 μM), HT (50 μM), and HTA (50 μM) for 48 h. (+ and − are indicative of DEX treatment only; the type of test inhibitor treatment is stated in the axis). Nuclear proteins extracted from treated BJ cultures were directly assayed. (B) Histone proteins extracted from treated cultures were analysed through Western blotting, where methylation status was determined via immunodetection via mono-methylated lysines on histone H3. Figure S5. Stimulation of MAO enzyme activity by HC within BJ cells. (A–C) The BJ cells were incubated with normal growth medium (−HC) or 10 μM

HC (+HC) for 5 days and treated with 50 µM OLP, 50 µM OLC, or 5 µM TCP for 24 h. The results depict the activity of (A) MAO-A and (B) MAO-B and (C) total MAO activity. The data obtained are represented as the mean ± SEM from duplicate assays. * $p \leq 0.05$, ** $p \leq 0.01$, *** $p \leq 0.001$, and **** $p \leq 0.0001$ quantified using a 2-way ANOVA with Tukey's post-hoc multiple comparisons test. Figure S6. Developmental pathway of neurons derived from hESCs. The cells were incubated with EGF and bFGF to induce neuron-like characteristics. Following neurosphere formation, the cells appeared as progenitors (+19 d) where half the total volume of NBM media from wells was replaced every 3 days to promote late neuronal differentiation and transformation into mature neurons. Figure S7. NMR spectra for the synthesised sample of OLP. OLP was dissolved in dDMSO and the NMR analysis was performed using a Bruker 400MHz Avance equipped with an iProbe. The results can be seen for the (A) ^{1}H, (B) ^{13}C, and (C) ^{13}C DEPT-135 experiments. Figure S8. LC-MS analysis for the synthesised sample of OLP. (A) OLP was dissolved in DMSO and 5 µL was injected into an HPLC-DAD-ESI-MS system (Agilent 1260 Infinity II LC MSD, Australia). (B) The product ions for the major (15.98 min) and (C-F) minor peaks (8.00, 23.45, 24.40, and 30.61 min) can be seen. The major peak contains the OLP ion (m/z = 283, and $2m/z$ = 567). Figure S9. Upregulation of MAO protein expression by HC and DEX within BJ cells. (A) The BJ cells were incubated with 1, 5, or 10 µM of HC for 5 days. (B) The BJ cells were incubated with 10, 50, or 100 µM of DEX for a period of 5 or 7 days. Untreated (N/T) cells received normal growth medium. Western blot results are shown, where MAO-A/B expression was measured through immunodetection. "+" and "−" denote the presence or absence of HC and DEX within cell culture groups, respectively. Figure S10. Potent inhibition of MAO enzyme expression by OLP and OLC within BJ cells. To stimulate MAO expression, the BJ cells were incubated with 10 µM HC or 100 µM DEX. Following 5 or 7 days of incubation with HC or DEX, respectively, the cells were washed and treated with 50 µM OLP, 50 µM OLC, or 5 µM TCP. Untreated cells received normal growth medium. Western blot results are shown, where MAO-A/B expression was measured through immunodetection. "+" and "−" denote the presence or absence of HC and DEX within cell culture groups, respectively.

Author Contributions: Conceptualisation, T.C.K., X.D., K.N., A.H. and A.E.-O.; data curation, K.V., J.J.L., E.P., S.L., S.M.B., V.X. and S.J.W.; formal analysis, K.V., J.J.L., E.P., S.L., S.M.B., V.X., S.J.W. and E.G.; methodology, T.C.K., X.D., K.N., A.H., E.G. and A.E.-O.; supervision, T.C.K., X.D., K.N., A.H. and A.E.-O.; writing—original draft, T.C.K., K.V., J.J.L., E.P., S.L., S.M.B., V.X., S.J.W., X.D., K.N., A.H., E.G. and A.E.-O.; writing—review and editing, T.C.K., K.V., J.J.L., E.P., S.L., S.M.B., V.X., S.J.W., X.D., K.N., A.H., E.G. and A.E.-O. All authors have read and agreed to the published version of the manuscript.

Funding: This research received no external funding.

Data Availability Statement: The data presented in this study are available on request from the corresponding author.

Acknowledgments: We would like to acknowledge intellectual and financial support provided by McCord Research (North Liberty, IA, USA). We would like to thank Ana Antonic Baker at the Department of Neuroscience, Monash University, for the provision of the hESC (H9) cell line and protocols for differentiation. A.E.O. is a National Health and Medical Research Council (NHMRC) Senior Research Fellow (grant 1154650) and acknowledges grant support from NHMRC Clinical Trials and Cohort Studies (grant 2014763). E.P. is supported by an Australian Government Research Training Program Scholarship. We thank the National Computing Infrastructure (NCI) and the Pawsey Supercomputing Centre in Australia (funded by the Australian Government). Furthermore, we thank the Spartan High Performance Computing service (University of Melbourne) and the Partnership for Advanced Computing in Europe (PRACE) for awarding access to Piz Daint, hosted at the Swiss National Supercomputing Centre (CSCS), Switzerland.

Conflicts of Interest: Author Erik Goebel was employed by company LLC. The remaining authors declare that the research was conducted in the absence of any commercial or financial relationships that could be construed as a potential conflict of interest.

References

1. GBD 2019 Mental Disorders Collaborators. Global, regional, and national burden of 12 mental disorders in 204 countries and territories, 1990–2019: A systematic analysis for the Global Burden of Disease Study 2019. *Lancet Psychiatry* **2022**, *9*, 137–150. [CrossRef] [PubMed]
2. Penninx, B.W.J.H.; Lamers, F.; Milaneschi, Y.S. 03.02—Clinical heterogeneity in major depressive disorder. *Eur. Neuropsychopharmacol.* **2018**, *28*, S59–S60. [CrossRef]
3. Goldberg, D. The heterogeneity of "major depression". *World Psychiatry* **2011**, *10*, 226–228. [CrossRef] [PubMed]
4. World Health Organization. *International Classification of Diseases for Mortality and Morbidity Statistics (11th Revision)*; World Health Organization: Geneva, Switzerland, 2018.
5. Depressive Disorders. In *Diagnostic and Statistical Manual of Mental Disorders*; American Psychiatric Association Publishing: Washington, DC, USA, 2022. [CrossRef]
6. Lee, S.; Jeong, J.; Kwak, Y.; Park, S.K. Depression research: Where are we now? *Mol. Brain* **2010**, *3*, 8. [CrossRef] [PubMed]
7. Pitsillou, E.; Bresnehan, S.M.; Kagarakis, E.A.; Wijoyo, S.J.; Liang, J.; Hung, A.; Karagiannis, T.C. The cellular and molecular basis of major depressive disorder: Towards a unified model for understanding clinical depression. *Mol. Biol. Rep.* **2020**, *47*, 753–770. [CrossRef]
8. Son, S.Y.; Ma, J.; Kondou, Y.; Yoshimura, M.; Yamashita, E.; Tsukihara, T. Structure of human monoamine oxidase A at 2.2-A resolution: The control of opening the entry for substrates/inhibitors. *Proc. Natl. Acad. Sci. USA* **2008**, *105*, 5739–5744. [CrossRef]
9. Bach, A.W.; Lan, N.C.; Johnson, D.L.; Abell, C.W.; Bembenek, M.E.; Kwan, S.W.; Seeburg, P.H.; Shih, J.C. cDNA cloning of human liver monoamine oxidase A and B: Molecular basis of differences in enzymatic properties. *Proc. Natl. Acad. Sci. USA* **1988**, *85*, 4934–4938. [CrossRef]
10. Binda, C.; Wang, J.; Pisani, L.; Caccia, C.; Carotti, A.; Salvati, P.; Edmondson, D.E.; Mattevi, A. Structures of human monoamine oxidase B complexes with selective noncovalent inhibitors: Safinamide and coumarin analogs. *J. Med. Chem.* **2007**, *50*, 5848–5852. [CrossRef] [PubMed]
11. Thase, M.E. The role of monoamine oxidase inhibitors in depression treatment guidelines. *J. Clin. Psychiatry* **2012**, *73* (Suppl. S1), 10–16. [CrossRef]
12. Tian, H.; Hu, Z.; Xu, J.; Wang, C. The molecular pathophysiology of depression and the new therapeutics. *MedComm* **2022**, *3*, e156. [CrossRef]
13. Liu, L.; Wang, H.; Chen, X.; Zhang, Y.; Zhang, H.; Xie, P. Gut microbiota and its metabolites in depression: From pathogenesis to treatment. *eBioMedicine* **2023**, *90*, 104527. [CrossRef] [PubMed]
14. Leonard, B.E. Inflammation and depression: A causal or coincidental link to the pathophysiology? *Acta Neuropsychiatr.* **2018**, *30*, 1–16. [CrossRef] [PubMed]
15. Mitsis, T.; Papakonstantinou, E.; Vlachakis, D. Biological mechanisms underlying depression, epigenetics and their interplay (Review). *Int. J. Epigen.* **2023**, *3*, 3. [CrossRef]
16. Wu, M.S.; Li, X.J.; Liu, C.Y.; Xu, Q.; Huang, J.Q.; Gu, S.; Chen, J.X. Effects of Histone Modification in Major Depressive Disorder. *Curr. Neuropharmacol.* **2022**, *20*, 1261–1277. [CrossRef] [PubMed]
17. Wu, F.; Zhou, C.; Yao, Y.; Wei, L.; Feng, Z.; Deng, L.; Song, Y. 3-(Piperidin-4-ylmethoxy)pyridine Containing Compounds Are Potent Inhibitors of Lysine Specific Demethylase 1. *J. Med. Chem.* **2016**, *59*, 253–263. [CrossRef] [PubMed]
18. Perillo, B.; Tramontano, A.; Pezone, A.; Migliaccio, A. LSD1: More than demethylation of histone lysine residues. *Exp. Mol. Med.* **2020**, *52*, 1936–1947. [CrossRef] [PubMed]
19. Binda, C.; Li, M.; Hubalek, F.; Restelli, N.; Edmondson, D.E.; Mattevi, A. Insights into the mode of inhibition of human mitochondrial monoamine oxidase B from high-resolution crystal structures. *Proc. Natl. Acad. Sci. USA* **2003**, *100*, 9750–9755. [CrossRef] [PubMed]
20. Allen, S.R.; Amrein, R.; Guentert, T.W.; Hartmann, D.; Lorscheid, T.; Schoerlin, M.P.; Vranesic, D. The Pharmacology of Reversible Monoamine Oxidase Inhibitors. *Br. J. Psychiatry* **1989**, *155*, 66–71. [CrossRef]
21. Corona, G.; Spencer, J.P.; Dessì, M.A. Extra virgin olive oil phenolics: Absorption, metabolism, and biological activities in the GI tract. *Toxicol. Ind. Health* **2009**, *25*, 285–293. [CrossRef]
22. Estruch, R.; Ros, E.; Salas-Salvadó, J.; Covas, M.-I.; Corella, D.; Arós, F.; Gómez-Gracia, E.; Ruiz-Gutiérrez, V.; Fiol, M.; Lapetra, J.; et al. Primary Prevention of Cardiovascular Disease with a Mediterranean Diet Supplemented with Extra-Virgin Olive Oil or Nuts. *N. Engl. J. Med.* **2018**, *378*, e34. [CrossRef]
23. Keys, A.; Keys, M. *How to Eat Well and Stay Well the Mediterranean Way*; Doubleday: New York, NY, USA, 1975.
24. Menotti, A.; Puddu, P.E. How the Seven Countries Study contributed to the definition and development of the Mediterranean diet concept: A 50-year journey. *Nutr. Metab. Cardiovasc. Dis.* **2015**, *25*, 245–252. [CrossRef]
25. Pett, K.D.; Willett, W.C.; Vartiainen, E.; Katz, D.L. The Seven Countries Study. *Eur. Heart J.* **2017**, *38*, 3119–3121. [CrossRef]
26. Psaltopoulou, T.; Sergentanis, T.N.; Panagiotakos, D.B.; Sergentanis, I.N.; Kosti, R.; Scarmeas, N. Mediterranean diet, stroke, cognitive impairment, and depression: A meta-analysis. *Ann. Neurol.* **2013**, *74*, 580–591. [CrossRef] [PubMed]
27. Jacka, F.N.; O'Neil, A.; Opie, R.; Itsiopoulos, C.; Cotton, S.; Mohebbi, M.; Castle, D.; Dash, S.; Mihalopoulos, C.; Chatterton, M.L.; et al. A randomised controlled trial of dietary improvement for adults with major depression (the 'SMILES' trial). *BMC Med.* **2017**, *15*, 23. [CrossRef] [PubMed]

28. Beauchamp, G.K.; Keast, R.S.J.; Morel, D.; Lin, J.; Pika, J.; Han, Q.; Lee, C.-H.; Smith, A.B.; Breslin, P.A.S. Ibuprofen-like activity in extra-virgin olive oil. *Nature* **2005**, *437*, 45–46. [CrossRef]
29. Bonvino, N.P.; Liang, J.; McCord, E.D.; Zafiris, E.; Benetti, N.; Ray, N.B.; Hung, A.; Boskou, D.; Karagiannis, T.C. OliveNet™: A comprehensive library of compounds from Olea europaea. *Database* **2018**, *2018*, bay016. [CrossRef]
30. Nan, J.N.; Ververis, K.; Bollu, S.; Rodd, A.L.; Swarup, O.; Karagiannis, T.C. Biological effects of the olive polyphenol, hydroxytyrosol: An extra view from genome-wide transcriptome analysis. *Hell. J. Nucl. Med.* **2014**, *17* (Suppl. 1), 62–69. [PubMed]
31. Gorzynik-Debicka, M.; Przychodzen, P.; Cappello, F.; Kuban-Jankowska, A.; Marino Gammazza, A.; Knap, N.; Wozniak, M.; Gorska-Ponikowska, M. Potential Health Benefits of Olive Oil and Plant Polyphenols. *Int. J. Mol. Sci.* **2018**, *19*, 686. [CrossRef] [PubMed]
32. Serreli, G.; Deiana, M. Biological Relevance of Extra Virgin Olive Oil Polyphenols Metabolites. *Antioxidants* **2018**, *7*, 170. [CrossRef]
33. Bianco, A.; Chiacchio, M.A.; Grassi, G.; Iannazzo, D.; Piperno, A.; Romeo, R. Phenolic components of Olea europea: Isolation of new tyrosol and hydroxytyrosol derivatives. *Food Chem.* **2006**, *95*, 562–565. [CrossRef]
34. Yin, W.; Löf, M.; Chen, R.; Hultman, C.M.; Fang, F.; Sandin, S. Mediterranean diet and depression: A population-based cohort study. *Int. J. Behav. Nut. Phys. Act.* **2021**, *18*, 153. [CrossRef] [PubMed]
35. Bizzozero-Peroni, B.; Martínez-Vizcaíno, V.; Fernández-Rodríguez, R.; Jiménez-López, E.; Núñez de Arenas-Arroyo, S.; Saz-Lara, A.; Díaz-Goñi, V.; Mesas, A.E. The impact of the Mediterranean diet on alleviating depressive symptoms in adults: A systematic review and meta-analysis of randomized controlled trials. *Nut. Rev.* **2024**, nuad176. [CrossRef]
36. Brown, E.S.; Chandler, P.A. Mood and Cognitive Changes During Systemic Corticosteroid Therapy. *Prim. Care Companion J. Clin. Psychiatry* **2001**, *3*, 17–21. [CrossRef]
37. Warrington, T.P.; Bostwick, J.M. Psychiatric Adverse Effects of Corticosteroids. *Mayo Clin. Proc.* **2006**, *81*, 1361–1367. [CrossRef]
38. Pitsillou, E.; Liang, J.; Hung, A.; Karagiannis, T.C. Chromatin modification by olive phenolics: In silico molecular docking studies utilising the phenolic groups categorised in the OliveNet™ database against lysine specific demethylase enzymes. *J. Mol. Graph. Model.* **2020**, *97*, 107575. [CrossRef] [PubMed]
39. Pitsillou, E.; Liang, J.; Hung, A.; Karagiannis, T.C. Molecular docking utilising the OliveNet™ library reveals novel phenolic compounds which may potentially target key proteins associated with major depressive disorder. *Comput. Biol. Chem.* **2020**, *86*, 107234. [CrossRef] [PubMed]
40. Niwa, H.; Sato, S.; Hashimoto, T.; Matsuno, K.; Umehara, T. Crystal Structure of LSD1 in Complex with 4-[5-(Piperidin-4-ylmethoxy)-2-(p-tolyl)pyridin-3-yl]benzonitrile. *Molecules* **2018**, *23*, 1538. [CrossRef]
41. Speranzini, V.; Rotili, D.; Ciossani, G.; Pilotto, S.; Marrocco, B.; Forgione, M.; Lucidi, A.; Forneris, F.; Mehdipour, P.; Velankar, S.; et al. Polymyxins and quinazolines are LSD1/KDM1A inhibitors with unusual structural features. *Sci. Adv.* **2016**, *2*, e1601017. [CrossRef]
42. Maiques-Diaz, A.; Somervaille, T.C. LSD1: Biologic roles and therapeutic targeting. *Epigenomics* **2016**, *8*, 1103–1116. [CrossRef]
43. Forneris, F.; Binda, C.; Adamo, A.; Battaglioli, E.; Mattevi, A. Structural Basis of LSD1-CoREST Selectivity in Histone H3 Recognition*. *J. Biol. Chem.* **2007**, *282*, 20070–20074. [CrossRef]
44. Edelstein, S.B.; Castiglione, C.M.; Breakfield, X.O. Monoamine oxidase activity in normal and Lesch-Nyhan fibroblasts. *J. Neurochem.* **1978**, *31*, 1247–1254. [CrossRef] [PubMed]
45. González-Acedo, A.; Ramos-Torrecillas, J.; Illescas-Montes, R.; Costela-Ruiz, V.J.; Ruiz, C.; Melguizo-Rodríguez, L.; García-Martínez, O. The Benefits of Olive Oil for Skin Health: Study on the Effect of Hydroxytyrosol, Tyrosol, and Oleocanthal on Human Fibroblasts. *Nutrients* **2023**, *15*, 2077. [CrossRef] [PubMed]
46. Cuyàs, E.; Castillo, D.; Llorach-Parés, L.; Lozano-Sánchez, J.; Verdura, S.; Nonell-Canals, A.; Brunet, J.; Bosch-Barrera, J.; Joven, J.; Valdés, R.; et al. Computational de-orphanization of the olive oil biophenol oleacein: Discovery of new metabolic and epigenetic targets. *Food Chem. Toxicol.* **2019**, *131*, 110529. [CrossRef]
47. Cuyàs, E.; Gumuzio, J.; Lozano-Sánchez, J.; Carreras, D.; Verdura, S.; Llorach-Parés, L.; Sanchez-Martinez, M.; Selga, E.; Pérez, G.J.; Scornik, F.S.; et al. Extra Virgin Olive Oil Contains a Phenolic Inhibitor of the Histone Demethylase LSD1/KDM1A. *Nutrients* **2019**, *11*, 1656. [CrossRef] [PubMed]
48. Zheng, Y.-C.; Shen, D.-D.; Ren, M.; Liu, X.-Q.; Wang, Z.-R.; Liu, Y.; Zhang, Q.-N.; Zhao, L.-J.; Zhao, L.-J.; Ma, J.-L.; et al. Baicalin, a natural LSD1 inhibitor. *Bioorganic Chem.* **2016**, *69*, 129–131. [CrossRef] [PubMed]
49. Abdulla, A.; Zhao, X.; Yang, F. Natural Polyphenols Inhibit Lysine-Specific Demethylase-1 in vitro. *J. Biochem. Pharmacol. Res.* **2013**, *1*, 56–63. [PubMed]
50. Wang, J.; Zhang, X.; Yan, J.; Li, W.; Jiang, Q.; Wang, X.; Zhao, D.; Cheng, M. Design, synthesis and biological evaluation of curcumin analogues as novel LSD1 inhibitors. *Bioorganic Med. Chem. Lett.* **2019**, *29*, 126683. [CrossRef] [PubMed]
51. Duan, Y.-C.; Guan, Y.-Y.; Zhai, X.-Y.; Ding, L.-N.; Qin, W.-P.; Shen, D.-D.; Liu, X.-Q.; Sun, X.-D.; Zheng, Y.-C.; Liu, H.-M. Discovery of resveratrol derivatives as novel LSD1 inhibitors: Design, synthesis and their biological evaluation. *Eur. J. Med. Chem.* **2017**, *126*, 246–258. [CrossRef]
52. Jia, G.; Cang, S.; Ma, P.; Song, Z. Capsaicin: A "hot" KDM1A/LSD1 inhibitor from peppers. *Bioorganic Chem.* **2020**, *103*, 104161. [CrossRef]
53. Melfi, F.; Carradori, S.; Angeli, A.; D'Agostino, I. Nature as a source and inspiration for human monoamine oxidase B (hMAO-B) inhibition: A review of the recent advances in chemical modification of natural compounds. *Expert Opin. Drug Discov.* **2023**, *18*, 851–879. [CrossRef]

54. Bester, E.; Petzer, A.; Petzer, J.P. Coumarin derivatives as inhibitors of d-amino acid oxidase and monoamine oxidase. *Bioorganic Chem.* **2022**, *123*, 105791. [CrossRef]
55. Myburg, T.; Petzer, A.; Petzer, J.P. The inhibition of monoamine oxidase by harmine derivatives. *Results Chem.* **2022**, *4*, 100607. [CrossRef]
56. Zhang, Z.; Hamada, H.; Gerk, P.M. Selectivity of Dietary Phenolics for Inhibition of Human Monoamine Oxidases A and B. *Biomed. Res. Int.* **2019**, *2019*, 8361858. [CrossRef] [PubMed]
57. *The PyMOL Molecular Graphics System*; Version 1.8; Schrödinger LLC: New York, NY, USA, 2015.
58. Kim, S.; Chen, J.; Cheng, T.; Gindulyte, A.; He, J.; He, S.; Li, Q.; Shoemaker, B.A.; Thiessen, P.A.; Yu, B.; et al. PubChem 2023 update. *Nucleic Acids Res.* **2023**, *51*, D1373–D1380. [CrossRef]
59. Morris, G.M.; Huey, R.; Lindstrom, W.; Sanner, M.F.; Belew, R.K.; Goodsell, D.S.; Olson, A.J. AutoDock4 and AutoDockTools4: Automated docking with selective receptor flexibility. *J. Comput. Chem.* **2009**, *30*, 2785–2791. [CrossRef]
60. Dallakyan, S.; Olson, A.J. Small-molecule library screening by docking with PyRx. *Methods Mol. Biol.* **2015**, *1263*, 243–250. [CrossRef] [PubMed]
61. O'Boyle, N.M.; Banck, M.; James, C.A.; Morley, C.; Vandermeersch, T.; Hutchison, G.R. Open Babel: An open chemical toolbox. *J. Cheminformatics.* **2011**, *3*, 33. [CrossRef]
62. Jendele, L.; Krivak, R.; Skoda, P.; Novotny, M.; Hoksza, D. PrankWeb: A web server for ligand binding site prediction and visualization. *Nucleic Acids Res.* **2019**, *47*, W345–W349. [CrossRef]
63. Trott, O.; Olson, A.J. AutoDock Vina: Improving the speed and accuracy of docking with a new scoring function, efficient optimization, and multithreading. *J. Comput. Chem.* **2010**, *31*, 455–461. [CrossRef]
64. *Schrödinger Release 2022-2: Maestro*; Schrödinger LLC: New York, NY, USA, 2022.
65. Humphrey, W.; Dalke, A.; Schulten, K. VMD: Visual molecular dynamics. *J. Mol. Graph.* **1996**, *14*, 33–38. [CrossRef]
66. Zhou, P.; Jin, B.; Li, H.; Huang, S.Y. HPEPDOCK: A web server for blind peptide-protein docking based on a hierarchical algorithm. *Nucleic Acids Res.* **2018**, *46*, W443–W450. [CrossRef] [PubMed]
67. Krissinel, E.; Henrick, K. Inference of macromolecular assemblies from crystalline state. *J. Mol. Biol.* **2007**, *372*, 774–797. [CrossRef] [PubMed]

Disclaimer/Publisher's Note: The statements, opinions and data contained in all publications are solely those of the individual author(s) and contributor(s) and not of MDPI and/or the editor(s). MDPI and/or the editor(s) disclaim responsibility for any injury to people or property resulting from any ideas, methods, instructions or products referred to in the content.
