# Supplementary material for: Identification and Evaluation of Olive Phenolics in the Context of Amine Oxidase Enzyme Inhibition and Depression: In Silico Modelling and In Vitro Validation
Source: Molecules. 2024 May 23;29(11):2446. doi: 10.3390/molecules29112446 (PMC11173677; doi:10.3390/molecules29112446)
Supplement: Supplementary file 1 [file molecules-29-02446-s001.zip › molecules-3018314-final.zip!/molecules-3018314-tex.zip!/Definitions/logo-ccby-eps-converted-to.pdf]

CC BY
