# Supplementary material for: Identification and Evaluation of Olive Phenolics in the Context of Amine Oxidase Enzyme Inhibition and Depression: In Silico Modelling and In Vitro Validation
Source: Molecules. 2024 May 23;29(11):2446. doi: 10.3390/molecules29112446 (PMC11173677; doi:10.3390/molecules29112446)
Supplement: Supplementary file 1 [file molecules-29-02446-s001.zip › molecules-3018314-final.zip!/molecules-3018314-tex.zip!/Definitions/molecules-3018314-g002.pdf]

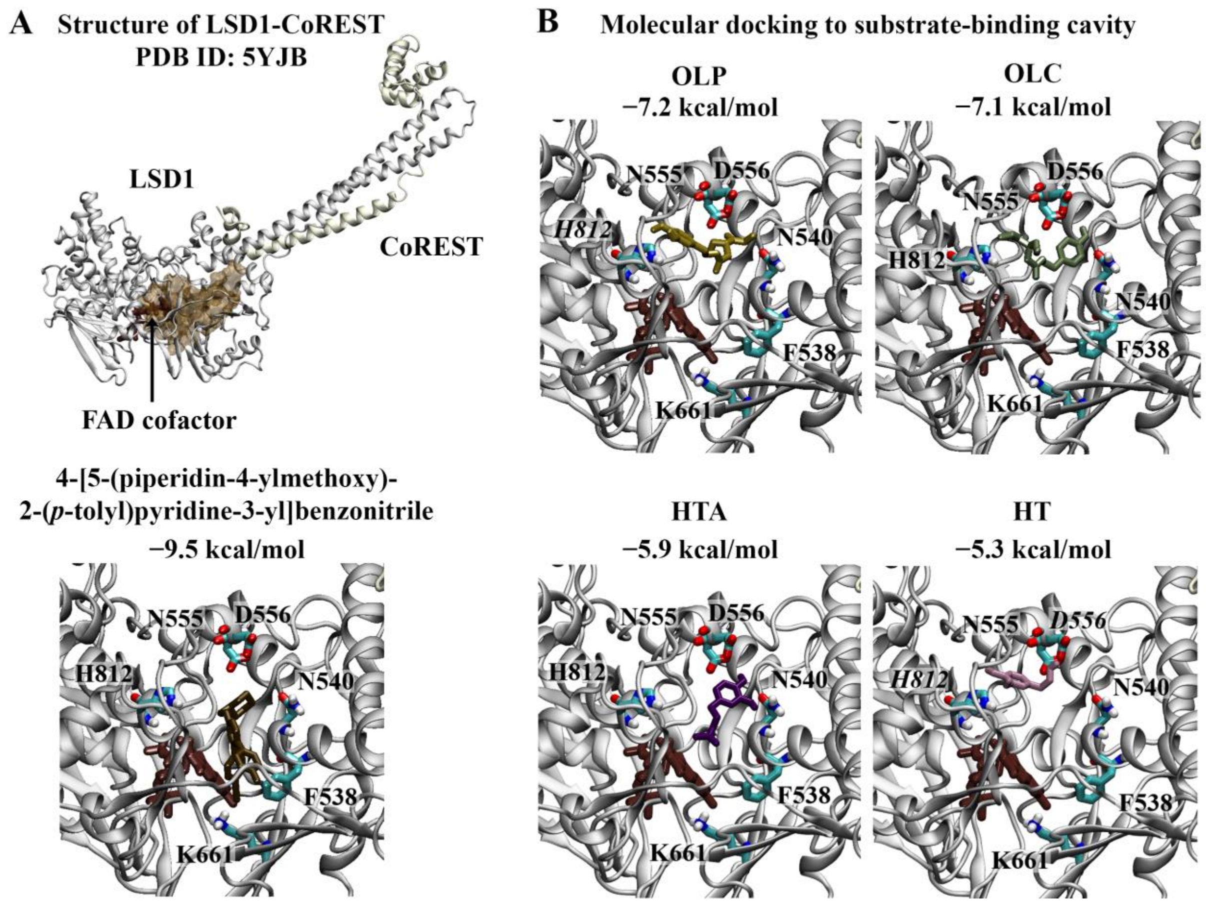
A
Structure of LSD1-CoREST
PDB ID: 5YJB
LSD1
CoREST
FAD cofactor
4-[5-(piperidin-4-ylmethoxy)-
2-(p-tolyl)pyridine-3-yl]benzonitrile
−9.5 kcal/mol
B
Molecular docking to substrate-binding cavity
OLP
−7.2 kcal/mol
OLC
−7.1 kcal/mol
HTA
−5.9 kcal/mol
HT
−5.3 kcal/mol
N555
D556
H812
N540
F538
K661
