# Supplementary material for: Identification and Evaluation of Olive Phenolics in the Context of Amine Oxidase Enzyme Inhibition and Depression: In Silico Modelling and In Vitro Validation
Source: Molecules. 2024 May 23;29(11):2446. doi: 10.3390/molecules29112446 (PMC11173677; doi:10.3390/molecules29112446)
Supplement: Supplementary file 1 [file molecules-29-02446-s001.zip › molecules-3018314-final.zip!/molecules-3018314-tex.zip!/Definitions/molecules-3018314-g003.pdf]

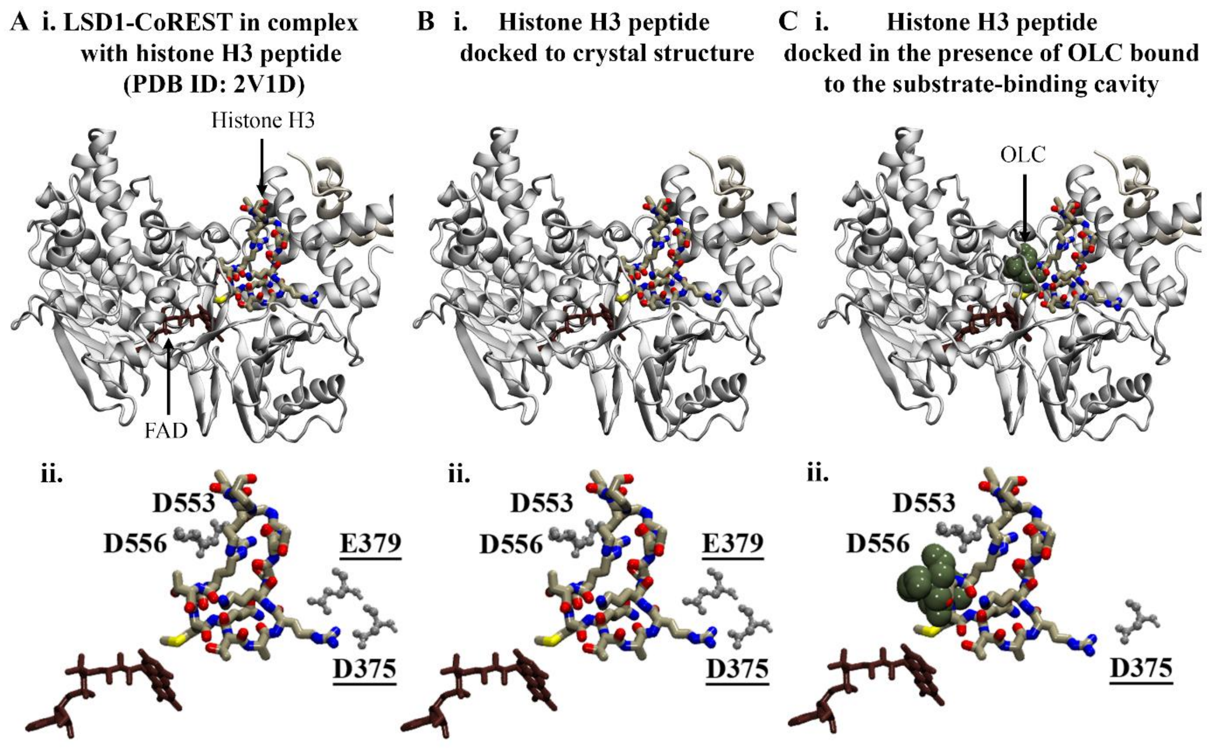
A i. LSD1-CoREST in complex with histone H3 peptide (PDB ID: 2V1D)
B i. Histone H3 peptide docked to crystal structure
C i. Histone H3 peptide docked in the presence of OLC bound to the substrate-binding cavity
Histone H3
FAD
OLC
ii.
D553
D556
E379
D375
ii.
D553
D556
E379
D375
ii.
D553
D556
D375
