# Supplementary material for: Identification and Evaluation of Olive Phenolics in the Context of Amine Oxidase Enzyme Inhibition and Depression: In Silico Modelling and In Vitro Validation
Source: Molecules. 2024 May 23;29(11):2446. doi: 10.3390/molecules29112446 (PMC11173677; doi:10.3390/molecules29112446)
Supplement: Supplementary file 1 [file molecules-29-02446-s001.zip › molecules-3018314-final.zip!/molecules-3018314-tex.zip!/Definitions/molecules-3018314-g004.pdf]

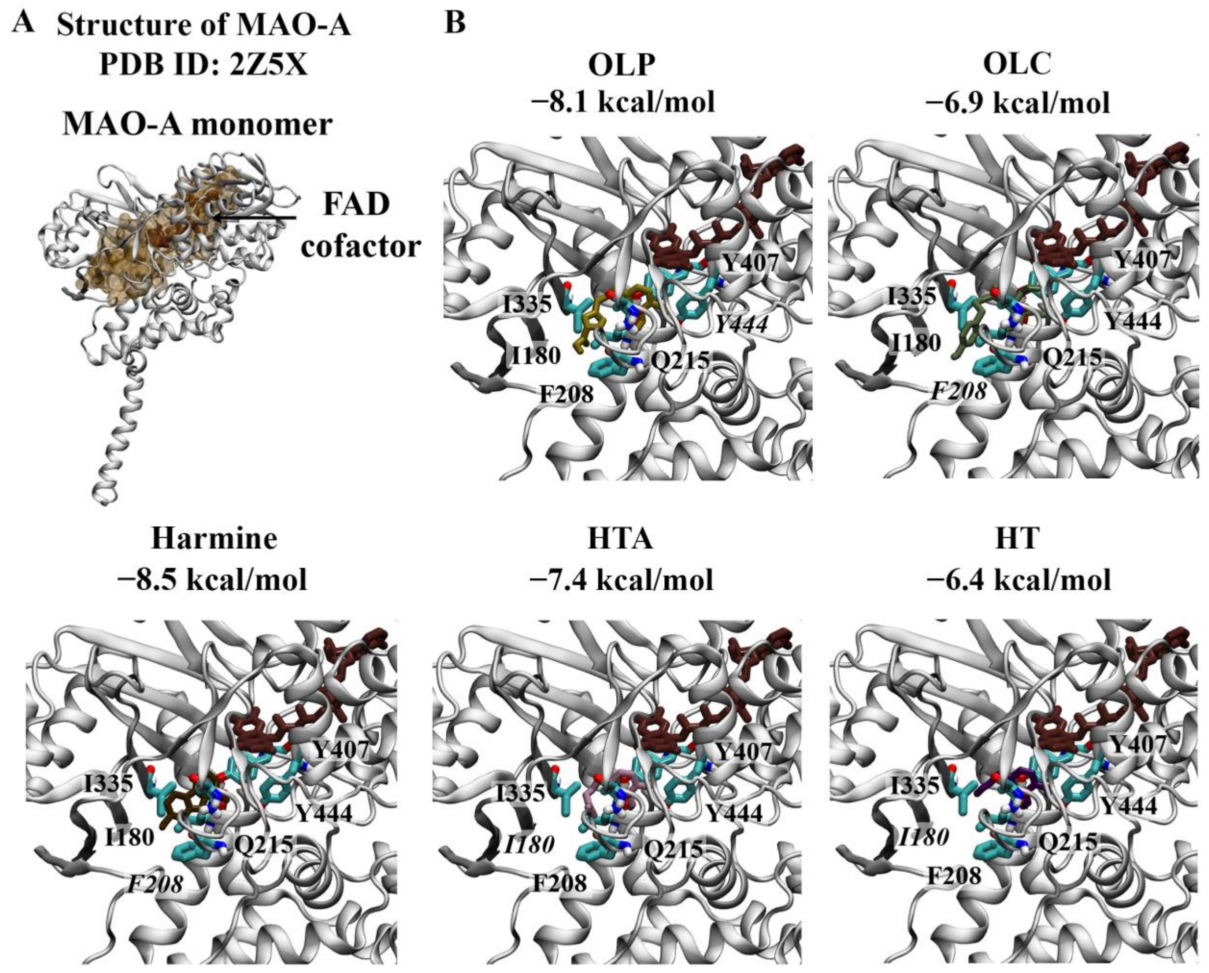
A
Structure of MAO-A
PDB ID: 2Z5X
MAO-A monomer
FAD cofactor
B
OLP
−8.1 kcal/mol
I335
I180
F208
Q215
Y407
Y444
OLC
−6.9 kcal/mol
I335
I180
F208
Q215
Y407
Y444
Harmine
−8.5 kcal/mol
I335
I180
F208
Q215
Y407
Y444
HTA
−7.4 kcal/mol
I335
I180
F208
Q215
Y407
Y444
HT
−6.4 kcal/mol
I335
I180
F208
Q215
Y407
Y444
