# Supplementary material for: Identification and Evaluation of Olive Phenolics in the Context of Amine Oxidase Enzyme Inhibition and Depression: In Silico Modelling and In Vitro Validation
Source: Molecules. 2024 May 23;29(11):2446. doi: 10.3390/molecules29112446 (PMC11173677; doi:10.3390/molecules29112446)
Supplement: Supplementary file 1 [file molecules-29-02446-s001.zip › molecules-3018314-final.zip!/molecules-3018314-tex.zip!/Definitions/molecules-3018314-g005.pdf]

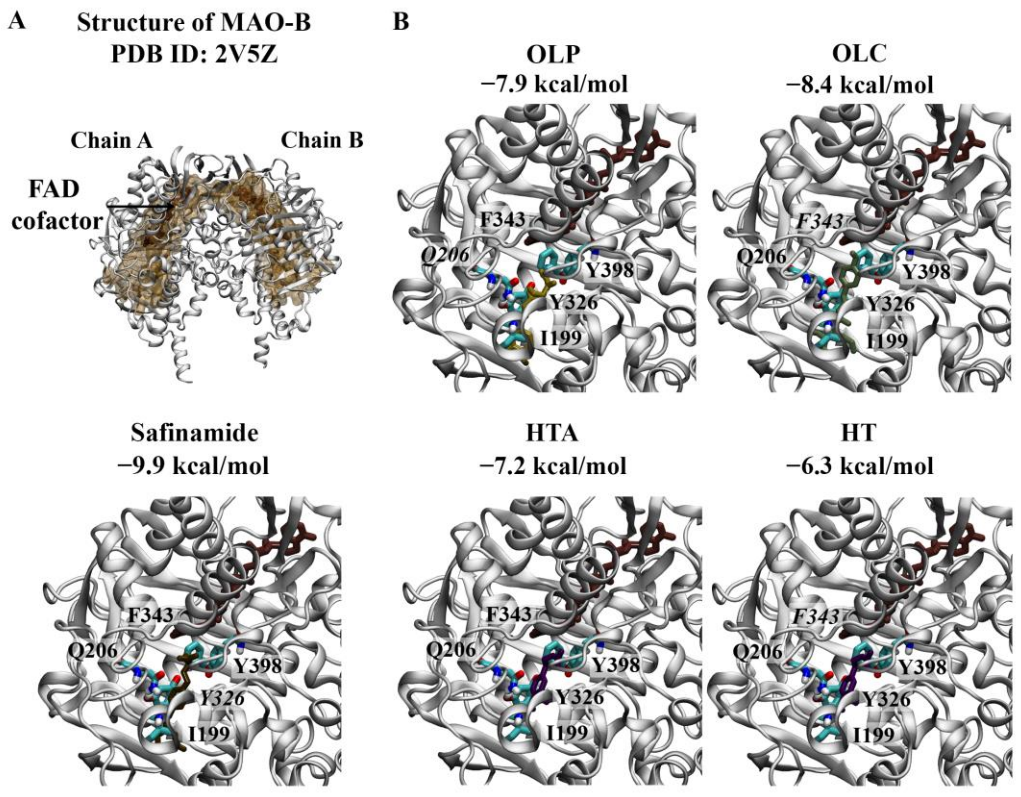
A
Structure of MAO-B
PDB ID: 2V5Z
Chain A
Chain B
FAD
cofactor
B
OLP
−7.9 kcal/mol
F343
Q206
Y398
Y326
I199
OLC
−8.4 kcal/mol
F343
Q206
Y398
Y326
I199
Safinamide
−9.9 kcal/mol
F343
Q206
Y398
Y326
I199
HTA
−7.2 kcal/mol
F343
Q206
Y398
Y326
I199
HT
−6.3 kcal/mol
F343
Q206
Y398
Y326
I199
