# Supplementary material for: Identification and Evaluation of Olive Phenolics in the Context of Amine Oxidase Enzyme Inhibition and Depression: In Silico Modelling and In Vitro Validation
Source: Molecules. 2024 May 23;29(11):2446. doi: 10.3390/molecules29112446 (PMC11173677; doi:10.3390/molecules29112446)
Supplement: Supplementary file 1 [file molecules-29-02446-s001.zip › molecules-3018314-final.zip!/molecules-3018314-tex.zip!/Definitions/molecules-3018314-g007.pdf]

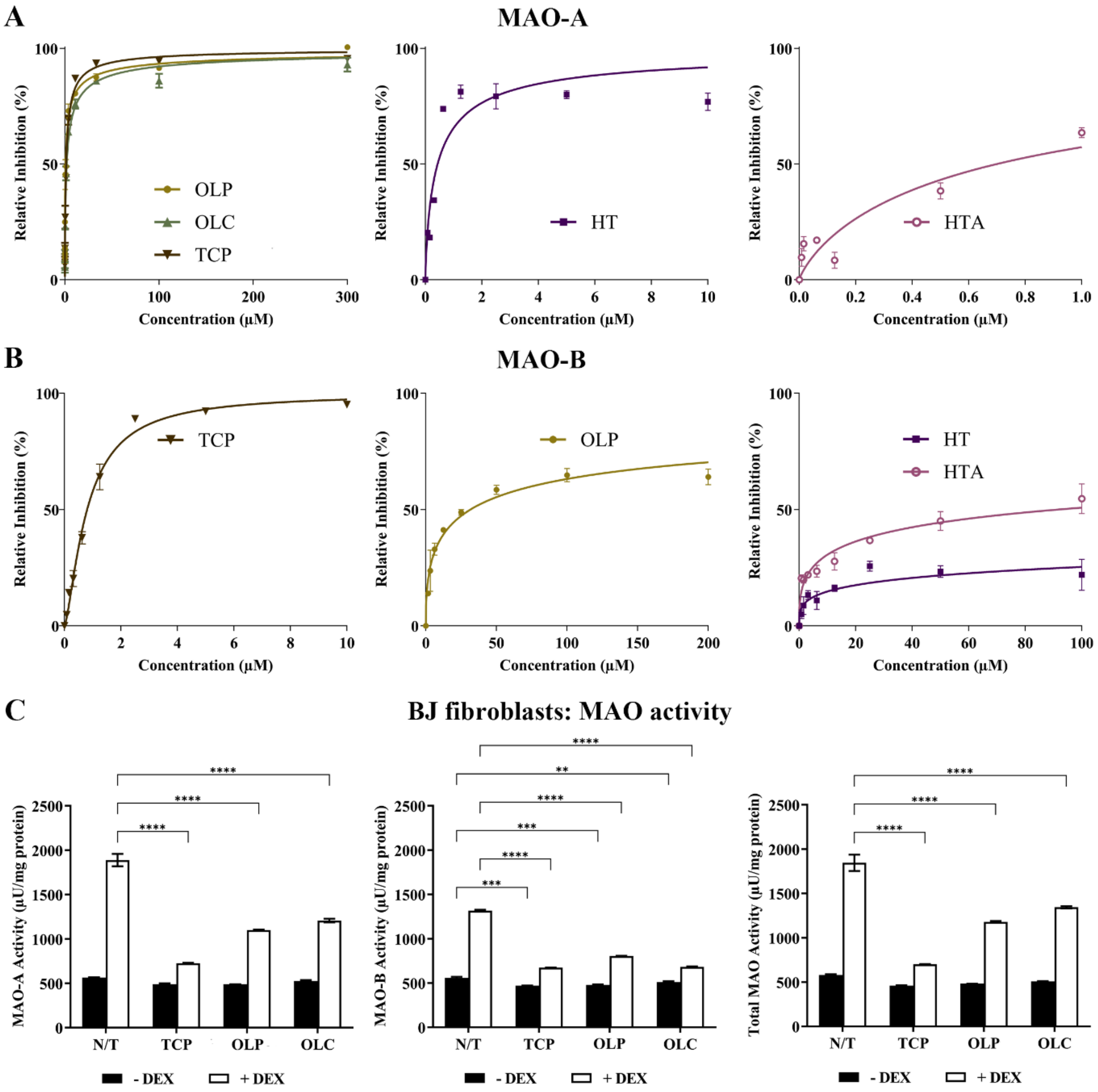
A
MAO-A
Relative Inhibition (%)
Concentration (µM)
OLP
OLC
TCP
HT
HTA
B
MAO-B
TCP
OLP
HT
HTA
C
BJ fibroblasts: MAO activity
MAO-A Activity (µU/mg protein)
MAO-B Activity (µU/mg protein)
Total MAO Activity (µU/mg protein)
N/T
TCP
OLP
OLC
- DEX
+ DEX
****
***
**
